# The malignant phenotype in breast cancer is driven by eIF4A1-mediated changes in the translational landscape

**DOI:** 10.1038/cddis.2014.542

**Published:** 2015-01-22

**Authors:** A Modelska, E Turro, R Russell, J Beaton, T Sbarrato, K Spriggs, J Miller, S Gräf, E Provenzano, F Blows, P Pharoah, C Caldas, J Le Quesne

**Affiliations:** 1 Department of Oncology and Cancer Research UK Cambridge Institute, University of Cambridge, Li Ka Shing Centre, Robinson Way, Cambridge CB2 0RE, UK; 2 Department of Haematology, University of Cambridge, NHS Blood and Transplant, Long Road, Cambridge CB2 0PT, UK; 3 Medical Research Council Toxicology Unit, Lancaster Road, Leicester LE1 9HN, UK; 4 School of Pharmacy, University of Nottingham, Nottingham NG7 2RD, UK; 5 Department of Medicine, University of Cambridge, Addenbrooke's Hospital, Hills Road, Cambridge CB2 0QQ, UK; 6 Cambridge Experimental Cancer Medicine Centre (ECMR) and NIHR Cambridge Biomedical Research Centre, Cambridge University Hospitals NHS Foundation Trust, Cambridge CB2 0QQ, UK; 7 Cambridge Breast Unit and Cambridge University Hospitals NHS Foundation Trust, Hills Road, Cambridge CB2 0QQ, UK; 8 Strangeways Research Institute, 2 Worts' Causeway, Cambridge CB1 8RN, UK

## Abstract

Human mRNA DeXD/H-box helicases are ubiquitous molecular motors that are required for the majority of cellular processes that involve RNA metabolism. One of the most abundant is eIF4A, which is required during the initiation phase of protein synthesis to unwind regions of highly structured mRNA that would otherwise impede the scanning ribosome. Dysregulation of protein synthesis is associated with tumorigenesis, but little is known about the detailed relationships between RNA helicase function and the malignant phenotype in solid malignancies. Therefore, immunohistochemical analysis was performed on over 3000 breast tumors to investigate the relationship among expression of eIF4A1, the helicase-modulating proteins eIF4B, eIF4E and PDCD4, and clinical outcome. We found eIF4A1, eIF4B and eIF4E to be independent predictors of poor outcome in ER-negative disease, while in contrast, the eIF4A1 inhibitor PDCD4 was related to improved outcome in ER-positive breast cancer. Consistent with these data, modulation of eIF4A1, eIF4B and PCDC4 expression in cultured MCF7 cells all restricted breast cancer cell growth and cycling. The eIF4A1-dependent translatome of MCF7 cells was defined by polysome profiling, and was shown to be highly enriched for several classes of oncogenic genes, including G-protein constituents, cyclins and protein kinases, and for mRNAs with G/C-rich 5′UTRs with potential to form G-quadruplexes and with 3′UTRs containing microRNA target sites. Overall, our data show that dysregulation of mRNA unwinding contributes to the malignant phenotype in breast cancer via preferential translation of a class of genes involved in pro-oncogenic signaling at numerous levels. Furthermore, immunohistochemical tests are promising biomarkers for tumors sensitive to anti-helicase therapies.

The malignant phenotype is the consequence of dysregulated gene expression. Most regulation occurs post-transcriptionally,^1^ and the major determinant of protein abundance is translational control.^2^

Translation initiation is rate limiting, highly regulated and dependent on the coordinated action of eukaryotic initiation factors (eIFs).^3, 4^ The DEAD-box helicase eIF4A1 is required to unwind structured RNA elements within the 5′ untranslated region (5′UTR) to facilitate ribosome binding and scanning, its activity is stimulated by interaction with the proteins eIF4B^5^ and eIF4E,^6^ and inhibited by the tumor suppressor PDCD4.^7^ The isoform eIF4A2 can also participate in translation initiation, but is also implicated in the function of microRNAs.^8^ Structured 5′UTRs are inhibitory to translation initiation,^9^ so alterations in helicase activity are expected to have message-specific effects.

Alterations in the expression of several eIF4A1 activity-modulating proteins have been observed in many cancers. eIF4E is a well-established oncogene,^10, 11^ and the translation of several oncogenic mRNAs with long or structured 5′UTRs, such as c-*myc*, cyclin D1 and ornithine decarboxylase (ODC) were shown to correlate with eIF4E expression.^12^ eIF4B is required for HeLa cell growth,^13^ and eIF4B overexpression was associated with poor survival of lymphoma patients, probably resulting from increased eIF4A1-dependent expression of DAXX, BCL2 and ERCC5.^14^ In contrast, high levels of PDCD4 were associated with a reduction in the expression of growth-promoting factors such as ODC and CDK4,^15^ and correlate with good outcome in a small study of ER-positive breast tumors,^16^ while levels were reduced in several tumor types including breast adenocarcinoma.^17^

Expression of eIF4A1 itself has not been widely investigated in tumors. However, the above findings and data from a number of model systems implicate eIF4A1 in the malignant phenotype. Inhibition of eIF4A results in reduced expression of several oncogenes, including cyclin D1, Bcl-x and MUC1,^18, 19^ and improves chemosensitivity in mouse models of lymphoma.^19, 20^ In breast and prostate cancer xenograft models, eIF4A inhibition enhanced apoptosis and diminished tumor angiogenesis and proliferation.^21^ Recently, several natural small molecules that inhibit eIF4A have been described, notably silvestrol^21^ and hippuristanol,^22^ and there is considerable interest in such agents as potential anti-cancer treatments.

Given the evidence that eIF4A1-mediated helicase activity may be a useful therapeutic target, we carried out a large-scale study to investigate the prevalence and clinical significance of the expression of eIF4A1 and its modulators in breast cancer, and found all these proteins to have striking relationships with survival. We went on to show that eIF4A1 activity is limiting for cell growth and cycling in cultured breast cancer cells. Then, in order to find mechanisms linking helicase activity to these phenotypes, we obtained the global eIF4A1-dependent translatome in cultured MCF7 cells, and demonstrated that numerous oncogenic mRNAs are directly translationally upregulated by eIF4A1. These mRNAs are GC rich, and enriched for motifs with the potential to form G-quadruplexes.

Our data show that diverse modes of increased eIF4A1 helicase activity expand the translatome to allow growth promotion, suggesting that eIF4A1 inhibitors could provide good therapeutic options for patients with tumors exerting this mechanism, and that simple immunohistochemical tests are promising biomarkers to predict sensitivity.

## Results

### Expression of pro-helicase factors predicts poor outcome in ER-negative disease

**Figure 1:**
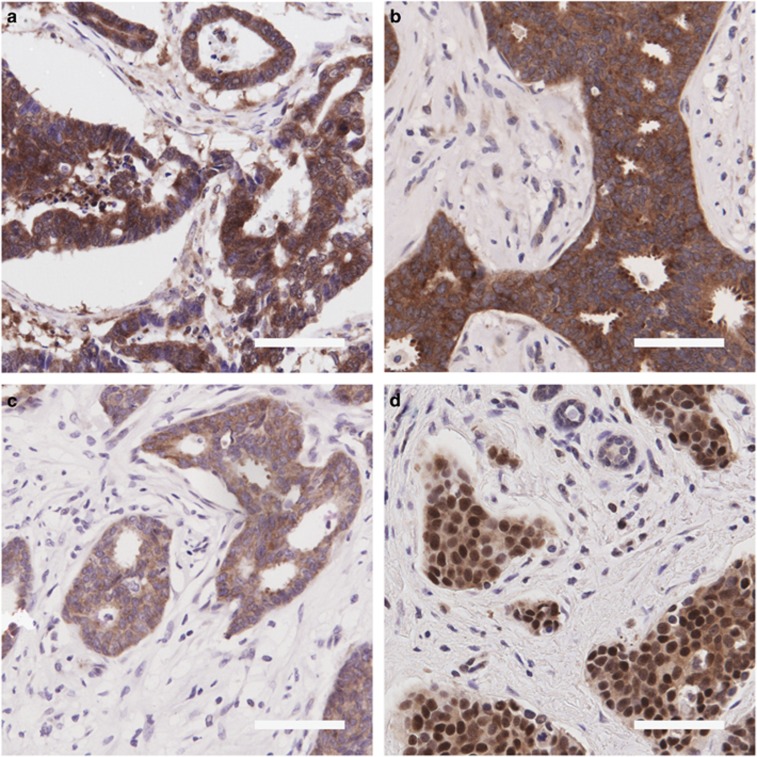
Representative immunohistochemistry images. Photomicrographs of representative TMA cores were taken. (**a**) eIF4E (strong cytoplasmic staining), (**b**) eIF4A1 (strong cytoplasmic staining), (**c**) eIF4B (moderate cytoplasmic staining) and (**d**) PDCD4 (strong nuclear staining and moderate cytoplasmic staining). Scale bar=100 *μ*m

**Figure 2:**
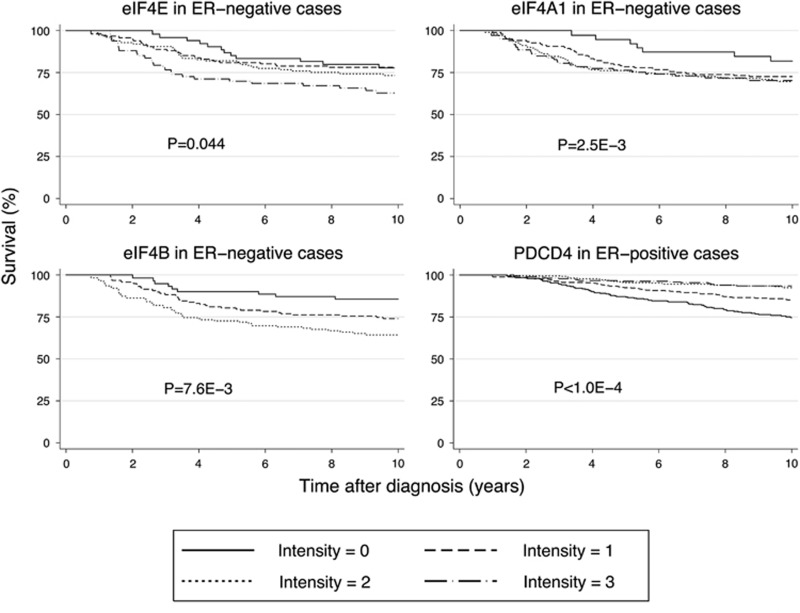
Kaplan-Meier graphs. Curves show survival of patient cohorts defined by their expression of factors that influence mRNA helicase activity as measured by immunohistochemistry. eIF4E, eIF4A1 and eIF4B stains are quantified in the cytoplasm, and PDCD4 in the nucleus. Numbers at risk are listed in Supplementary Table 9. *P*-values refer to the log-rank test for trend across expression categories, calculated over 10 years in all cases except for eIF4A1; this variable shows time dependence and the test is calculated over the first 3 years only

To assess eIF4A1 helicase activity in breast tumors, tissue microarrays (TMAs) derived from 3903 patients were scored for eIF4E, eIF4A1, eIF4B and PDCD4 (summarized in Table 1 and Supplementary Table 1). Representative images are shown in Figure 1. When incorporated into univariate Cox survival models, eIF4E, eIF4A1 and eIF4B all predict poor outcome in ER-negative cases only (Figure 2 and Supplementary Table 2). Only eIF4A1 showed a significant time-dependent component. Multivariate models were constructed to further test prognostic value. In ER-negative disease modeled with all three pro-helicase factors, eIF4B maintained significance (Table 2, model 1). When included in a model with all key clinicopathological markers (HR=1.5, *P*=0.021; model 2). When parsimonized by stepwise removal of non-significant variables, the final model contained only eIF4B (HR=1.7, *P*=0.002), HER2 and nodal status (model 3). eIF4A1 had similar relationships when substituted into the same model (model 4).

### PDCD4 expression predicts good outcome in ER-positive disease

The endogenous eIF4A1 inhibitor PDCD4 predicts good outcome in ER-positive disease when quantified in either the nucleus or the cytoplasm, although nuclear PDCD4 performs better in a bivariate model with both compartments (Table 2, model 5). In a model with all key clinicopathological parameters including the proliferation marker aurora kinase A, PDCD4 retained significance (HR=0.7, *P*<0.001; model 6). In the parsimonized model, PDCD4 retained this hazard ratio alongside grade, nodal metastasis and proliferation (models 7 and 8).

### Association of translation initiation factors with PDCD4 and ER

eIF4E, eIF4A1 and eIF4B were associated (Spearman's *ρ*=0.31–0.34), possibly reflecting coregulation (Supplementary Table 3). Nuclear and cytoplasmic PDCD4 were also associated (*ρ*=0.41) reflecting the known nucleocytoplasmic shuttling of this protein.^23^ PDCD4 correlates with ER expression, especially nuclear PDCD4 (*ρ*=0.35).

### Associations with clinicopathological variables

eIF4A1 and eIF4B were associated with higher histological grade (*P*<0.001 and *P*=0.004) in ER-negative tumors (Supplementary Table 4A and 4B). In ER-positive tumors, PDCD4 was associated with lower grade, smaller size and less nodal metastasis (*P*≤0.001).

### eIF4A1, eIF4B and PDCD4 influence breast cancer cell proliferation and cell cycle

**Figure 3:**
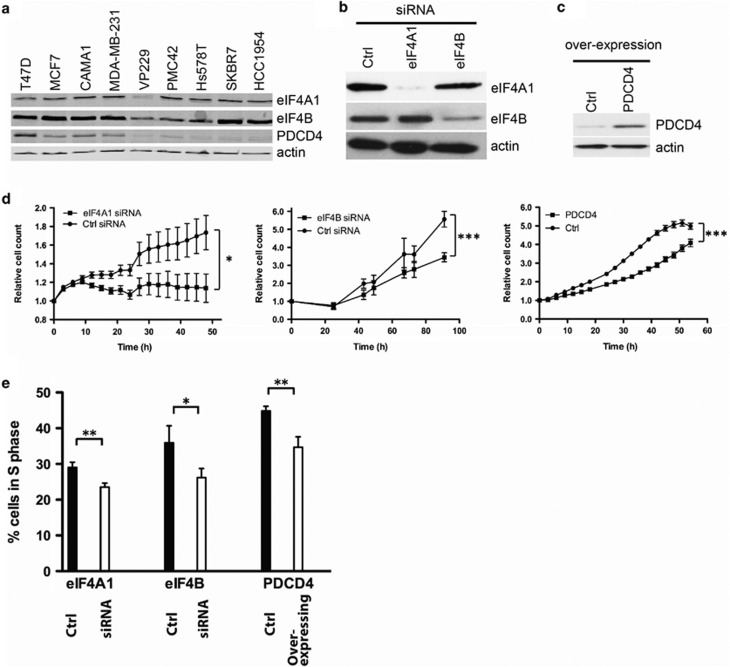
mRNA helicase activity reduction decreases MCF7 cell proliferation by interfering with the progression through cell cycle. (**a**) Immunoblot analysis of endogenous eIF4A1, eIF4B and PDCD4 protein expression in nine breast cancer cell lines. (**b**) Immunoblot analysis of eIF4A1 and eIF4B expression showing the efficacy of siRNA knockdown. (**c**) Immunoblot analysis of PDCD4 expression showing the overexpression of PDCD4 in stably transfected MCF7 cells. (**d**) Growth curves for cultured MCF7 cells treated with anti-eIF4A1 or anti-eIF4B siRNA or control siRNA, or cells stably overexpressing PDCD4 and control cells. Growth curves were obtained by confluency measurements using IncuCyte (for eIF4A1 knockdown and PDCD4 overexpression) or counting cells using ViCell (for eIF4B knockdown). *Points*, mean value of at least a triplicate for a typical experiment; significance indicated by *P<0.05 and ***P<0.001, as determined by two-way ANOVA. (**e**) Flow cytometric analysis of BrdU and PI incorporation to determine percentage of cells in S phase on eIF4A1 or eIF4B knockdown or PDCD4 overexpression. Data are representative of two biological replicates including at least one technical duplicate. Significance indicated by *P<0.05 and **P<0.01, as determined by *t*-test

We then sought causative relationships among mediators of eIF4A1 helicase function, cell growth and the cell cycle by screening a panel of breast cancer cell lines (Figure 3a). MCF7 cells expressed high levels of eIF4A1 and moderate levels of eIF4B and PDCD4, and was chosen as a model cell line.

eIF4A1 and eIF4B were transiently decreased using siRNA, and PDCD4 was overexpressed using a lentiviral vector (Figures 3b and c). Growth curves were generated for control and treated cells (Figure 3d), and the data show that decreased helicase activity significantly slowed down cellular proliferation.

The proportion of cells in S phase of the cell cycle was determined (Figure 3e) and in all cases decreased helicase activity was associated with a reduction of the S-phase fraction, indicating that eIF4A1 activity favours entry into S phase.

### A subset of mRNAs is dependent on eIF4A1 for efficient translation

**Figure 4:**
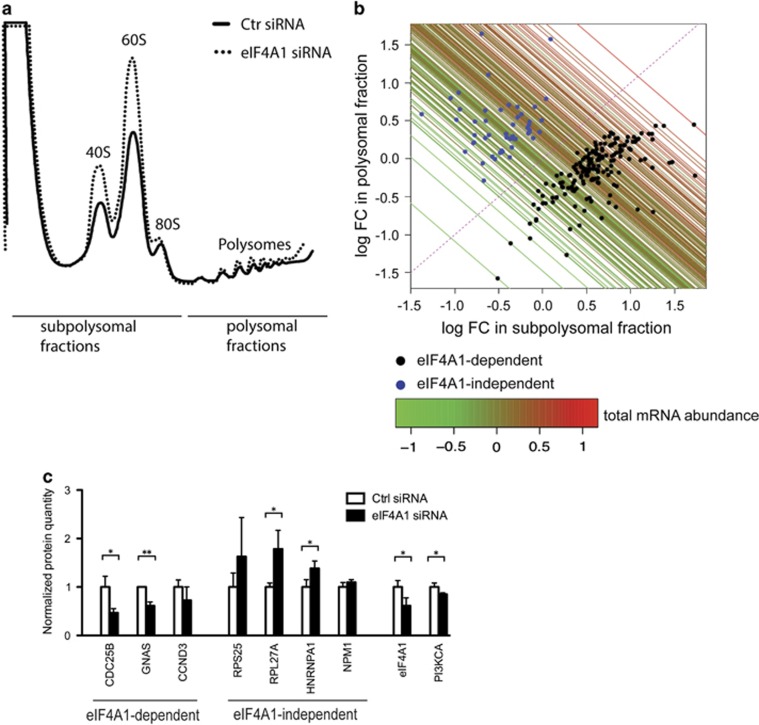
A subset of mRNAs is dependent on eIF4A1 for efficient translation. MCF7 cells were transfected with either control siRNA or anti-eIF4A1 siRNA and analyzed 48 h post-transfection. (**a**) Polysome analysis of RNA. Ribosomes were stalled with cycloheximide and mRNAs separated according to their ribosome load by sucrose-gradient centrifugation. The positions of the 40S, 60S and 80S ribosomal subunits and polysomal peaks are indicated, as well as fractions pooled for subsequent mRNA analysis. (**b**) Identification of eIF4A1-dependent and -independent messages. mRNAs from pools indicated in control and eIF4A1 knockdown MCF7 cells were quantified by RNA sequencing (the experiment was performed in quadruplicate). The Bayesian model was applied to identify mRNAs that show significant shifts between polysomes and subpolysomes following eIF4A1 knockdown. Each mRNA identified was plotted according to log-fold change in the subpolysomal and polysomal fractions; mRNAs below *y*=*x* are translationally downregulated (eIF4A1-dependent mRNAs) and mRNAs above *y*=*x* show the reverse tendency (eIF4A1 independent). Colored lines drawn through each mRNA plot point show the change in the separately quantified total mRNA abundance. This confirms that in general the modeled changes in fractionated mRNAs are reflected at the total mRNA level, that is, an mRNA seen to go up in both subpolysomal and polysomal fractions generally shows an increased total abundance. (**c**) Immunoblot analysis of protein levels of eIF4A1-dependent and -independent messages. Translationally upregulated proteins increase at the protein level and vice versa. The experiment was performed in triplicate. Significance indicated by *<0.05 and ***<0.01

We then sought to define the eIF4A1-dependent translatome. Sucrose-gradient ultracentrifugation was used to isolate total, polysomal and subpolysomal mRNA fractions from control cells or cells where eIF4A1 levels were reduced by siRNA. The decreased eIF4A1 expression resulted in a small increase in free ribosomal subunits, consistent with slight global reduction in protein synthesis (Figure 4a).

RNAseq libraries were generated and sequenced by Illumina HiSeq (Illumina, San Diego, CA, USA), and transcript and gene expression levels were estimated using MMSEQ.^24^ To validate the quantification method, we compared levels from RNA sequencing with estimates obtained using microarray analysis of the same biological starting material. Overall, results from the two methods correlated strongly (Supplementary Figure 1; median Spearman's *ρ*=0.70), especially for highly expressed genes. Reduced correlation for genes with low expression is consistent with a reduced dynamic range for microarrays due to non-specific hybridization. Our subsequent analyses utilized the RNAseq data only.

To identify helicase-dependent (i.e., more polysomal/translated in the presence of eIF4A1) and -independent (i.e., the inverse tendency) mRNAs, a recently described Bayesian model selection method, MMDIFF,^25^ was employed on the RNAseq data (Figure 4b and Supplementary Table 5). One hundred seventy-five eIF4A1-dependent mRNAs that shift from polysomes to subpolysomes following eIF4A1 knockdown were identified, as were 49 mRNAs which show paradoxical translational upregulation following knockdown. To validate these effects, we examined protein levels of several genes identified as eIF4A1 dependent or independent by immunoblotting (Figure 4c). We also tested the effect of eIF4A1 knockdown on the oncogene PI3KCA; although not identified in our screen PI3KCA that harbors a known driving mutation in MCF7 cells.^26^ PI3KCA was also seen to be eIF4A1 dependent at the protein level.

### eIF4A1 dependence is related to 5′UTR structure

**Figure 5:**
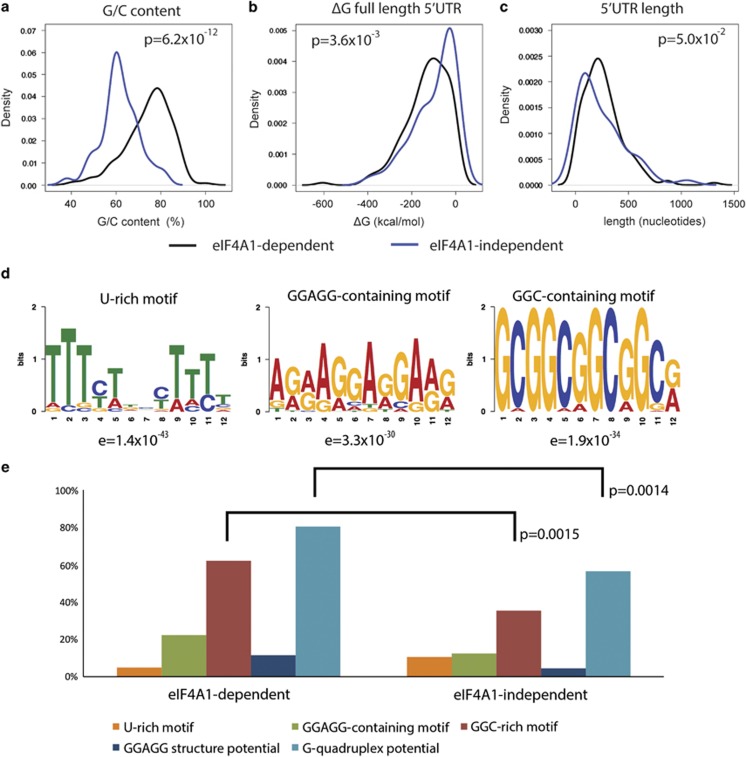
eIF4A1-dependent and -independent mRNAs have qualitatively different 5′UTRs. eIF4A1-dependent and -independent mRNA 5′UTRs were characterized in terms of (**a**) G/C content, (**b**) predicted folding stability and (**c**) 5′UTR length. *P*-values were calculated using Kolmogorov-Smirnov test. (**d**) Recurrent sequence motifs were discovered in the eIF4A-dependent 5′UTRs. (**e**) GGC-containing motifs were enriched in eIF4A-dependent sequences, as was the predicted potential to form classical G-quadruplexes

Since eIF4A1 is believed to unwind structures in 5′UTR mRNA, we sought properties of 5′UTRs associated with eIF4A1 dependence (Figure 5 and Supplementary Table 6). G/C content proved to be the most significant determining factor (Figure 5a; *P*=6.2e–12). The predicted minimum free-folding energy of the whole 5′UTR (ΔG) showed a significant but weaker relationship (*P*=3.6e–3; Figure 5b). There was no significant relationship with 5′UTR length (Figure 5c) or the presence of upstream AUG start codons.

We went on to seek enriched motifs up to 12-nt long using the MEME motif elicitation software^27^ within the eIF4A1-dependent messages, as compared to scrambled sequence. The most significantly identified motifs were a U-rich tract (*e*-value=1.4e–43; Figure 5d), a G/A-rich motif including the consensus GGAGG (*e*=3.3e–30), and a G/C-rich motif of the form GC(GGC)_3_G (*e*=1.9e–34).

When we examined the eIF4A1-dependent and eIF4A1-independent groups for the numbers of UTRs that contain the identified motifs, we found that the (GGC)-repeat motif was enriched in the eIF4A1-dependent group (62 *versus* 35%, *P*=0.0015; Figure 5e). The G/A-rich motif was even more enriched in this group (22 *versus* 13%) but did not meet nominal significance (*P*=0.15). The U-rich motif in contrast showed the opposite trend (5 *versus* 10%, *P*=0.31).

The (GGC)_n_ repeat motif is highly suggestive of G-quadruplex formation, and (GGC)_4_ RNA repeats have recently been shown to have G-quadruplex–forming properties.^28^ The GGAGG motif also has higher-order folding potential; two GGAGG motifs connected by a short linker can fold to form a compact structure containing a G-quadruplex, and two of these can form a dimeric RNA G-quadruplex structure *in trans.*^29^

We therefore interrogated the sequences of the eIF4A-dependent and eIF4A-independent 5′UTRs for the potential to form these structures. The eIF4A-dependent group is relatively enriched for G-quadruplex–forming sequences (80 *versus* 36%, *P*=0.0014) as well as paired GGAGG motifs (12 *versus* 4%, *P*=0.17; Figure 5e).

### eIF4A1 dependence identifies genes with diverse roles in the malignant phenotype

We examined the eIF4A1-dependent and -independent subsets of mRNAs (Figure 4b) for statistically over-represented structural protein families using GeneTrail^30^ (Supplementary Table 7). The group of 175 eIF4A1-dependent transcripts is enriched for three structural protein families: G-protein *α*-subunits (*P*=3.9e–6), cyclin N-terminal domains (*P*=3.1e–3) and serine-threonine protein kinases (*P*=3.2e-3). We noticed that this group also contained several other genes involved in oncogenic signaling pathways, such as TGFB1, SMAD2, ARAF and the CDK1 cyclin activator CDC25B.

We then analyzed a list of all genes ranked by eIF4A1 dependence by gene set enrichment analysis (GSEA)^31^ (Supplementary Table 7). This identified numerous signaling pathways, including IP3/calcium, FGFR, EGFR and HER2 pathways (FDR *q*-value near to 0), as well as MAP kinase (*q*=0.022) and TGF-beta pathways (*q*=0.071). This was accompanied by association with gene-ontological (GO) categories such as amino acid phosphorylation (*q*=0.055) and chromatin modification (FDR=0.092). In addition, several malignant KEGG categories were enriched including colorectal (*q*=1.1e–3), endometrial (*q*=2.1e–3) and renal cell carcinomas (*q*=0.032).

### eIF4A1-independent genes are related to DNA repair and apoptosis

The list of 49 eIF4A1-independent genes shows over-representation of zinc finger proteins (*P*=0.010). In GSEA analysis, the eIF4A1-independent arm was enriched for the ATM DNA damage pathway (*q*=0.015), alongside annotations for DNA damage resulting in apoptosis (*q*=0.11) and ribosomal proteins (*q*=0.078).

### eIF4A1 dependence is related to the presence of miRNA target sites

We then tested for enrichment of mRNAs with predicted 3′UTR miRNA target sites. This large list of genes accounting for almost half the annotated mRNAs was highly enriched for eIF4A1 dependence (*q*=6.9e–3).

## Discussion

### Markers of helicase activity as prognostic and predictive biomarkers in breast cancer

We showed that the expression of proteins that drive or inhibit the unwinding of 5′UTRs was strongly predictive of outcome in breast cancer, in ways that were independent of known influential variables. This suggested that dysregulation of translational control affected tumor biology in other, unmodeled ways.

Expression of eIF4A1 and its stimulatory partners eIF4E and eIF4B all predicted poor outcome in ER-negative disease only. eIF4A1 and eIF4B predicted poor outcome in ER-negative tumors independently of lymph node status, which is the most influential single predictor of outcome in this group. This is unusual and potentially clinically useful. First, there are very few independent prognostic factors apart from lymph node status reported in these aggressive and pharmacologically intractable tumors, rare exceptions being a transcriptional signature related to immunity^32^ and the stem cell marker integrin alpha-6.^33^ Second, it indicates that helicase activity affects more than just proliferation, as proliferation is known to have little if any relationship with survival in ER-negative cases.^34^ Third, these proteins hold promise as biomarkers to direct anti-helicase therapy.

In ER-positive disease, both nuclear and cytoplasmic PDCD4 expression predicted good outcome, with moderate and high-expressing cases being at less than one-third the hazard of tumors without detectable protein, even after taking known predictive variables into consideration. This further suggests that any survival benefit of PDCD4 expression and consequent helicase inhibition is not solely due to effects on cellular proliferation.

There is a great need for biomarkers to identify patients with ER-positive tumors who are at low risk of recurrence and who might be spared unnecessary chemotherapy, and PDCD4 expression shows potential in this role. Low PDCD4 levels might also identify candidates for anti-helicase therapy.

Subcellular location of PDCD4 was of little relevance to its value as a prognostic factor (Table 2). However, nuclear PDCD4 displaced cytoplasmic PDCD4 from a bivariate survival model and it had somewhat different clinicopathological correlations. The significance of this is not clear, particularly as PDCD4 is known to shuttle, but might be related to putative additional nuclear functions of the protein.^35^

### Helicase activity and cell growth

All manipulations of eIFs and PDCD4 intended to decrease helicase activity caused diminished proliferation and entry of cells in S phase, consistent with previous findings regarding the individual inhibition of eIF4A1 and eIF4B in leukemia^19^ and HeLa cells.^13^ In addition, our finding that we could replicate the inhibitory effect on cell growth by overexpressing PDCD4 was consistent with the known inhibitory effect of PDCD4 on eIF4A1 function, and supports a role for PDCD4 expression in restricting tumor growth.

This suggests that multiple convergent mechanisms might be exploited by cancer cells to influence helicase activity thereby achieving similar dysregulatory and phenotypic effects.

### The eIF4A1-dependent translatome

A cohort of mRNAs was seen to be reliant on eIF4A1 expression for their effective translation, and a smaller group showed surprising recruitment into polysomes when eIF4A1 levels were decreased. These shifts were validated at the mRNA level by parallel microarray data, and at the protein level by immunoblotting.

The 175 helicase-dependent mRNAs encode a range of proteins implicated in oncogenesis, many of which are involved in intracellular signaling pathways. Therefore upregulation of helicase activity would be expected to be advantageous to a malignant clone. Conversely, inhibition of eIF4A1 would be expected to result in ‘dampening' of the same pathways with widespread normalizing effects upon the malignant phenotype.

In contrast, the group of 49 eIF4A1-independent mRNAs is enriched for pathways related to DNA damage detection and induction of apoptosis, and are less structured and G/C rich. They present a paradox; how can reduced mRNA helicase activity result in their translational upregulation? This is likely to be the result of enhanced availability of other components of the initiation machinery due to the reduced translation of eIF4A-dependent mRNAs. The observed enrichment for ribosomal protein mRNAs is consistent with this model, as they generally have short 5′UTRs containing terminal oligopyrimidine tracts (TOPs) with little potential for structure formation.

These findings enable us to construct a model for how dysregulation of translation initiation contributes to the malignant phenotype in breast cancer. Upregulation of pro-helicase activity proteins (eIF4A1, eIF4E and eIF4B) or downregulation of PDCD4 all lead to enhanced mRNA unwinding, which results in the specific translational upregulation of numerous proteins involved in cell signaling and proliferation. The result is enhanced signaling through multiple major mitogenic and oncogenic signaling pathways, favouring growth, entry into S phase and other aspects of the malignant phenotype in many ways. The detailed description of these mechanisms will require further work.

### Unwinding of G-quadruplexes due to enhanced eIF4A activity implicated in the phenotype of solid malignancy

We found that eIF4A1-dependent mRNAs have 5′UTRs with higher G/C content and greater predicted stability of secondary structure. Furthermore, they were enriched for sequence motifs containing (GGC)*n* motifs, and for predicted G-quadruplex–forming sequences. In addition, a GGAGG-containing element suggests further capacity for novel structure formation; these elements can form stable stacked structures of GGAGGA hexads and GGGG tetrads, and can do so in *trans*.^29^

These findings are complementary to a recent study of changes in translation brought about by eIF4A inhibition in a cultured T-cell acute lymphoblastic leukemia cell line.^28^ In that study, use of a non-specific eIF4A inhibitor in a cell line expressing high levels of eIF4A2 also led to translational downregulation of mRNAs enriched for G-quadruplex–forming sequences. The different roles of eIF4A1 and eIF4A2 are the subject of current debate, but taken together with our study this suggests that in some circumstances eIF4A1 and eIF4A2 may both be involved in the malignant phenotype via their capacity to unwind G-quadruplex structures. Our findings also, critically, extend this mechanism to epithelial malignancy and show relationships between eIF4A expression, the expression of endogenous eIF4A cofactors and inhibitors, and phenotypes at the cellular and patient survival levels.

### Translation initiation as a nexus for dysregulation of gene expression in several cancers

A recent study showed that maintenance of eIF4F, the complex containing eIF4A activated by eIF4E via its interaction with eIF4G^6^ was the common route for the acquisition of resistance to therapeutic drugs targeting mutant BRAF in melanoma cell lines and patients.^36^ Furthermore, eIF4A inhibitors showed synergistic effects on cellular proliferation in combination with anti-BRAF therapy.

Another study recently showed that predicted 5′UTR structure correlated with the presence of miRNA-binding sites in the 3′UTR, and was necessary to enable miRNA function^8^ with the implication that helicase dependence may be central to the miRNA mechanism. These data are consistent with our finding that eIF4A1-dependent mRNAs are highly enriched for miRNA target sequences. These same genes would be expected to be dysregulated by 3′UTR shortening and global miRNA loss, both of which are reported in a number of malignancies. We suggest that all three of these mechanisms (miRNA loss, 3′UTR shortening and helicase upregulation) contribute to malignant behavior by the derepression of the same group of eIF4A1-dependent mRNAs. Further characterization of these genes and the similarities between these modes of dysregulation will add significantly to our understanding of the malignant phenotype.

Taken with our findings that measures of eIF4A activity in breast tumor tissue are predictive of outcome, these studies provide further evidence that enhanced helicase activity is a key determinant of the malignant phenotype in a diverse range of malignancies. The eIF4G complex in general, and eIF4A mRNA helicase activity in particular, represent one of the most promising new therapeutic targets in many human cancers, and the way is clear for further studies and clinical trials.

## Materials and Methods

### Study populations and tissue arrays

The Study of Epidemiology and Risk Factors in Cancer Heredity (SEARCH) was used as the basis for immunohistochemical studies.^37^ In total, 3903 patients were included (summarized in Table 1). TMAs derived from this population have been used in several previous studies.^33, 34, 38^

### Immunohistochemistry

Immunostaining was performed using a Bond polymer refine kit and a Leica Bond-max autostainer (Supplementary Table 8). Immunohistochemical data for ER and aurora kinase A expression have been previously described in detail.^34, 38, 39^ Negative controls were performed simultaneously. Assay specificity was checked using cytoblocks prepared from cultured cells with siRNA knockdown (Supplementary Figure 2). Nine from a total of 118 arrays stained were deemed technical failures and excluded.

### Imaging and scoring

Core images were collected using an Ariol image capture system (Applied Imaging Corp, San Jose, CA, USA), and examined without knowledge of clinicopathological data. All markers displayed only minor variability between tumor cells, so staining intensity alone was recorded. Scores were given for the predominant intensity of staining, on a scale of 0–3 (0=negative, 1=weak, 2=moderate and 3=strong). eIF4B was scored on a scale of 0–2 due to relatively reduced dynamic range.

In total, 59% of cores provided data (8156 of 13 803). Missing data were due to cores ‘cutting out', as many of the arrays have been extensively used, poor core adherence or inadequate tumor cellularity.

### Statistical methods

Lymph node status was divided into 0, 1–3 and 4, or more positive regional lymph nodes. Tumor stage was divided into TNM stage 0, stages 1–2 and stages 3–4. Tumor size was divided into <2, 2–4.9 and >4.9 cm. When ordinal immunohistochemical variables were binarized, the cutoff was set at the first category above 0 that showed nominally significant prognostic value in a univariate Cox model. Associations between ordinal variables were quantified by Spearman's rank correlation with Pearson's chi-squared test. Other associations were tested using Fisher's exact test or Pearson's chi-squared test. The study complies with the REMARK (reporting recommendations for tumor-marker prognostic studies) criteria.^40^ STATA/SE13 (Statacorp, College Station, TX, USA) was used for statistical analyses.

### Cox regression survival modeling

The proportional-hazards assumption was verified by inspection of log-log plots. eIF4A1, eIF4B, eIF4E and PDCD4 were modeled as ordinal values, as all four variables showed incremental unidirectional changes in hazard ratio in univariate survival models had positive log-rank trend tests across categories (Figure 2). The measured outcome was breast cancer-related death, and survival analysis was restricted to 10 years after entry into the SEARCH study. Compensation was made for left truncation of data.^41^ Numbers at risk in Kaplan-Meier analyses are listed in Supplementary Table 9.

### Cell lines and cell culture

All cell lines used were authenticated in 2011–2012 using Promega GenePrint10 STR profiling kit (Promega Corporation, Madison, WI, USA). The cell lines T47D, MCF7, CAMA1, SKBR7 and MDA-MB-231 were grown in DMEM, HCC1954 and PMC42 were grown in RPMI, Hs578T was grown in DMEM supplemented with 10 *μ*g/ml insulin, and VP229 were grown in DMEM F12. All media were supplemented with 10% FBS (Life Technologies, Carlsbad, CA, USA).

### Knockdown and overexpression

Control ON-TARGETplus Non-targeting Pool and ON-TARGETplus SMARTpool siRNAs targeting eIF4E, eIF4A1 or eIF4B were transfected into cells according to manufacturer's instructions (Thermo Scientific, Waltham, MA, USA) using DharmaFECT 1 transfection reagent (Thermo Scientific; eIF4A1) or Lipofectamine RNAi MAX (Life Technologies; eIF4E and eIF4B).

High-titer viral particles including Precision LentiORF RFP control and Precision LentiORF PDCD4 clone were purchased from GE Healthcare (Little Chalfont, Buckinghamshire, UK) and used according to manufacturer's instructions to generate a cell line stably overexpressing PDCD4.

### Immunoblotting

Blots were probed using the following primary antibodies: anti-eIF4E (Santa Cruz Biotechnology, Santa Cruz, CA, USA; sc-9976), anti-eIF4A1 (Abcam, Cambridge, UK; ab31217), anti-eIF4B (Epitomics, Burlingame, CA, USA; 2232-1), anti-PDCD4 (Abcam, ab80590), anti-actin (Abcam, ab6276), CCND3 (Cell Signaling, Beverly, MA, USA; 2936), PI3KCA (Cell Signaling, 4249), CDC25B (Cell Signaling, 9525), NPM1 (Cell Signaling, 3542), GNAS (Abcam, ab83735), RPL27A (Abcam, ab74731), hnRNPA1 (Abcam, ab4791) and RPS25 (GeneTex, Irvine, CA, USA; 101526). Suitable secondary antibodies were used for chemiluminescence detection. Samples were analyzed from at least two independent experiments.

Films were scanned on an ImageScanner III using LabScan software (GE Healthcare) and proteins were quantified using ImageQuant software (GE Healthcare), or for Licor analysis, IRDye 680LT-conjugated secondary antibody (LI-COR Biosciences, Lincoln, NE, USA) was used, followed by scanning on the Odyssey system (LI-COR Biosciences).

### Cell proliferation assays

Cell number was determined either by direct counting using a ViCell XR cell viability analyzer (Beckman Coulter, Miami, FL, USA) using trypan blue cell viability assay or by monolayer confluence readings as calculated from images acquired by Essen IncuCyte (Essen BioScience, Ann Arbor, MI, USA). All data are a mean of at least three replicates.

### Cell cycle analysis

The fraction of proliferating cells in S phase was determined by bromodeoxyuridine (BrdU) incorporation. Cells were incubated with 10 *μ*M BrdU (Sigma-Aldrich, St. Louis, MO, USA) in normal culture medium for 1 h, fixed, washed, resuspended in denaturing solution for 20 min at room temperature, washed and resuspended in 0.5-ml neutralizing solution for 2 min, and washed again. BrdU incorporation was determined using a BrdU flow kit (BD Pharmingen, Franklin Lakes, NJ, USA). Washed cells were stained with propidium iodide and incubated for 1 h in darkness. Flow cytometric analysis was performed on a BD FACSCalibur flow cytometer with the CellQuest Pro software (BD Biosciences, Franklin Lakes, NJ, USA). The data were analyzed using FlowJo software (TreeStar, Inc., Ashland, OR, USA).

### Sucrose density gradient polysome profiling

Cytoplasmic lysates were fractionated to produce polysomal and subpolysomal populations of mRNAs as described previously.^14^ Pooled mRNAs were purified using a miRNeasy kit (Qiagen, Valencia, CA, USA).

### Gene expression microarrays

Total, subpolysomal and polysomal RNA was analyzed using Illumina gene expression microarrays (Human H12 v4 Beadchips; Illumina) according to the manufacturer's instructions. Data were processed using a number of Bioconductor packages.^42^ The beadarray package^43, 44^ was used for adjustment for spatial artifacts and quality assessment, and LOWESS normalization was applied to log_2_ transformed data. A probe-wise linear model was fitted to the data using Limma^45^ and the empirical Bayes method was used to identify statistically significant differentially expressed genes. The Benjamini-Hochberg method was used to control the false discovery rate.^46^ Hierarchical clustering of samples was based on Euclidean distance and complete linkage using the hclust function in R.

### RNA sequencing analysis

RNA sequencing libraries were created using Illumina TruSeq RNA sample preparation kit v2 (Illumina), and paired-end sequenced on a HiSeq 2000 to a length of 100 bp, using SBS chemistry version 3 from Illumina.

The RNAseq reads were aligned to version 68 of the Ensembl human reference cDNA and ncRNA sequences using Bowtie 1^47^ allowing for multi-mapping between reads and transcripts. The MMSEQ gene expression analysis software^24^ was used to estimate transcript expression levels. To perform a like-for-like comparison to microarray estimates, the MMSEQ marginal posteriors for the set of Ensembl 68 transcripts mapped to by each Illumina BeadChip probe were collapsed. Similarly, MMSEQ collapses marginal posteriors for the set of transcripts belonging each gene, thus providing gene-level expression estimates. The marginal posterior mean and S.D. of the log expression parameter corresponding to each transcript or set of transcripts (i.e., gene or probe) was then used as the outcome in a Bayesian model selection algorithm implemented in the MMDIFF software.^25^ The competing models are regression based and thus they can accommodate complex experimental designs. For differential expression analysis, we compared models using the following design matrices:


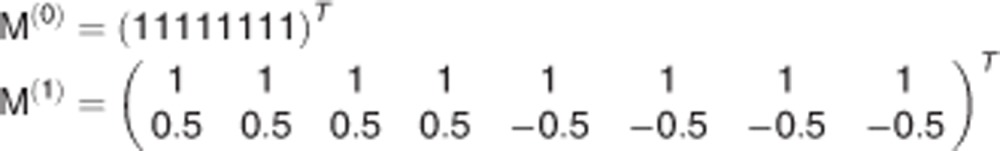


Where the first four rows correspond to one condition (e.g., knockdown samples in total unfractionated mRNA) and the last four rows correspond to another condition (e.g., control samples in the total unfractionated mRNA). The matrices were transposed to optimize the use of space.

In order to assess whether the log-fold change in expression between control and knockdown samples differed between subpolysomes and polysomes (i.e., a difference-of-difference analysis), models were compared using the following design matrices:


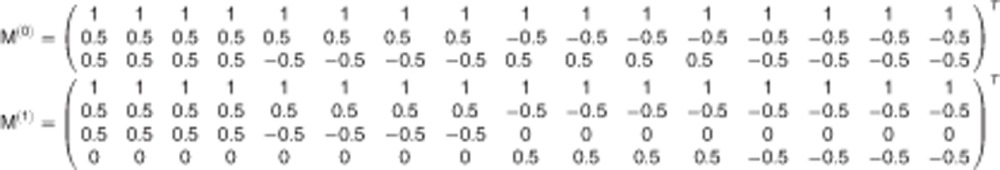


Where the rows correspond to subpolysomal RNA (eIF4A1 siRNA), subpolysomal RNA (control siRNA), polysomal RNA (eIF4A1 siRNA) and polysomal RNA (control siRNA), respectively in consecutive sets of four replicates.

In both sets of model comparisons, the prior distributions for the intercepts and other coefficients were set as described previously,^25^ while the probability of the more complex model being true was set to 10%. The posterior probability of the more complex model or, equivalently, a Bayes factor, was used as the basis for preferring the more complex model (differential expression or difference of difference, respectively) to the simpler model.

As the competing models are regression based, they may include, for instance, both an eIF4A1 siRNA *versus* ctrl siRNA effect and a polysomal *versus* subpolysomal effect on expression. To identify helicase-dependent mRNA transcripts, the simple model assumed that the log-fold change between subpolysomal (eIF4A1 siRNA) and subpolysomal (control siRNA) was the same as between polysomal (eIF4A1 siRNA) and polysomal (control siRNA), while the more complex model allowed the log-fold changes to differ. A prior probability of 0.1 that the complex model was true was specified and initially thresholded liberally on a posterior probability of 0.2 that the complex model was true in order to declare a transcript helicase dependent.

Our confidence in using this liberal threshold was increased by the strong correlation seen between modeled shifts between changes in polysomal and subpolysomal mRNA levels and total mRNA levels (Figure 4b), by reflection of predicted changes in translation at the protein level (Figure 4c), and by the strong relationship seen between helicase dependence and 5′UTR G/C content (Figure 5a).

### 5′UTR sequence analysis

The 5′UTR sequences of 84888 protein-coding transcripts were obtained from Ensembl Genome Browser (version 68) using the Ensembl Perl API.^48^ Minimum free energies (ΔG) of 5′UTR secondary structures were calculated using the Vienna RNA package (version 1.8.5).^49^ Kolmogorov-Smirnov test was performed to investigate whether there were significant differences between eIF4A1-dependent and -independent groups.

Sequence motifs within the eIF4A-dependent group of transcripts were discovered using the MEME suite of tools.^27, 50^ Where more than one transcript from one gene was present, only the longest UTR sequence was used; 156 sequences were used in the analysis. Motifs up to 12-nt long were sought in the 5′UTR sequences against the same sequences randomized using DREME. Frequencies of discovered motifs in eIF4A1-dependent and -independent mRNAs were assessed using the FLAG algorithm. G-quadruplex–forming potential was defined as the presence within the 5′UTR sequence of GG*GG*GG*GG or GGG*GGG*GGG*GGG where * is 1–7 of any nucleotide sequence. GGAGG-motif–forming potential was defined as the presence of GGAGG*GGAGG where * is 4–10 of any nucleotide.

### Gene annotation analyses

Over-representation analyses of eIF4A1-dependent and -independent groups were performed using GeneTrail.^30^ GSEA was performed using the Broad Institute package.^31, 51^ For GSEA, the list of transcripts was ranked by eIF4A1 dependence calculated as posterior probability x sign of shift between polysomal and subpolysomal fractions (Supplementary Figure 3). The list was rendered non-redundant by selection of the isoform with the highest posterior probability. Predicted miRNA targets were downloaded from TargetScanHuman (release 6.2).^52^

## Figures and Tables

**Table 1:** Study population summary

**Variable**		
Mean age (range in years)		53 (24–73)
Mean follow-up (range in years)		9.9 (0.6–20.2)
Number of breast cancer deaths (%)		582 (15)
5-Year survival (%)		3316 (85)
	**Category**	**Number (%)**
Age at diagnosis	≤55	2330 (60)
	>55	1573 (40)
	Missing	0
Grade	1	753 (19)
	2	1625 (42)
	3	1077 (28)
	Missing	448 (11)
Nodes	0	2182 (56)
	1–3	979 (25)
	>3	370 (9)
	Missing	372 (10)
Size	<20 mm	2083 (53)
	20–49 mm	1465 (38)
	≥50 mm	132 (3)
	Missing	223 (6)
ER	Negative	772 (20)
	Positive	2287 (59)
	Missing	844 (22)
HER2	Negative	2558 (66)
	Positive	349 (9)
	Missing	996 (26)

**Table 2:** Multivariate Cox regression survival models

	**Model (person-years of follow-up)**	**Variable**	***n* (deaths)**	**HR (95% CI)**	***P***	**T HR (95% CI)**	**T P**
ER-Negative	1 (1571)	eIF4E	259 (63)	1.2 (0.9–1.5)	0.291		
		eIF4A1		1.9 (0.9–4.0)	0.083	0.6 (0.3–0.9)	0.024
		eIF4B		1.6 (1.0–2.4)	0.037		
		Grade	292 (70)	1.1 (0.7–1.7)	0.554		
	2 (1796)	Nodes		2.1 (1.5–2.9)	<.001		
		Size		1.1 (0.7–1.6)	0.759		
		HER2		1.8 (1.0–2.9)	0.03		
		eIF4B		1.5 (1.1–2.1)	0.021		
	3 (2083)	Nodes	338 (74)	2.2 (1.6–2.9)	<0.001		
		HER2		1.7 (1.0–2.8)	0.039		
		eIF4B		1.7 (1.2–2.4)	0.002		
	4 (2853)	Nodes	466 (108)	2.4 (1.9–3.1)	<0.001		
		HER2		1.2 (1.0–2.4)	0.03		
		eIF4A1		2.1 (1.2–3.6)	0.006	0.6 (04–0.9)	0.017
ER-Positive	5 (10 686)	PDCD4(nuc)	1584 (171)	0.6 (0.5–0.7)	<0.001		
		PDCD4(cyt)		0.8 (0.6–1.0)	0.104		
	6 (6789)	Grade	967 (110)	1.5 (1.1–2.1)	0.008		
		Nodes		2.3 (1.8–3.0)	<0.001		
		Size		1.3 (0.9–1.8)	0.143		
		HER2		1.6 (0.9–2.6)	0.08		
		Aurora kinase		1.3 (1.1–1.6)	0.008		
		PDCD4(nuc)		0.7 (0.6–0.8)	<.001		
	7 (7402)	Grade	1044 (117)	1.6 (1.2–2.1)	0.004		
		Nodes		2.5 (1.9–3.1)	<0.001		
		Aurora kinase		1.3 (1.1–1.6)	0.005		
		PDCD4(nuc)		0.7 (0.5–0.8)	<0.001		
	8 (7402)	Grade	1044 (117)	1.8 (1.3–2.4)	<0.001		
		Nodes		2.5 (1.9–3.1)	<0.001		
		Aurora kinase		1.3 (1.1–1.6)	0.009		
		PDCD4(cyt)		0.7 (0.5–0.9)	0.003		

T HR and T P refer to the time-varying components of models incorporating eIF4A1

## Abbreviations

BrdU: bromodeoxyuridine
eIF: eukaryotic initiation factor
ER: estrogen receptor
ΔG: minimum free-folding energy
GO: gene ontology
GSEA: gene set enrichment analysis
HR: hazard ratio
miRNA: microRNA
ODC: ornithine decarboxylase
PDCD4: programmed cell death 4
SEARCH: Study of Epidemiology and Risk Factors in Cancer Heredity
TMA: tissue microarray
TOP: terminal oligopyrimidine tract
UTR: untranslated region
