# Supplementary material for: The malignant phenotype in breast cancer is driven by eIF4A1-mediated changes in the translational landscape
Source: Cell Death Dis. 2015 Jan 22;6(1):e1603–. doi: 10.1038/cddis.2014.542 (PMC4669741; doi:10.1038/cddis.2014.542)
Supplement: Supplementary Figures [file cddis2014542x1.pdf]

Supplementary Figure 1

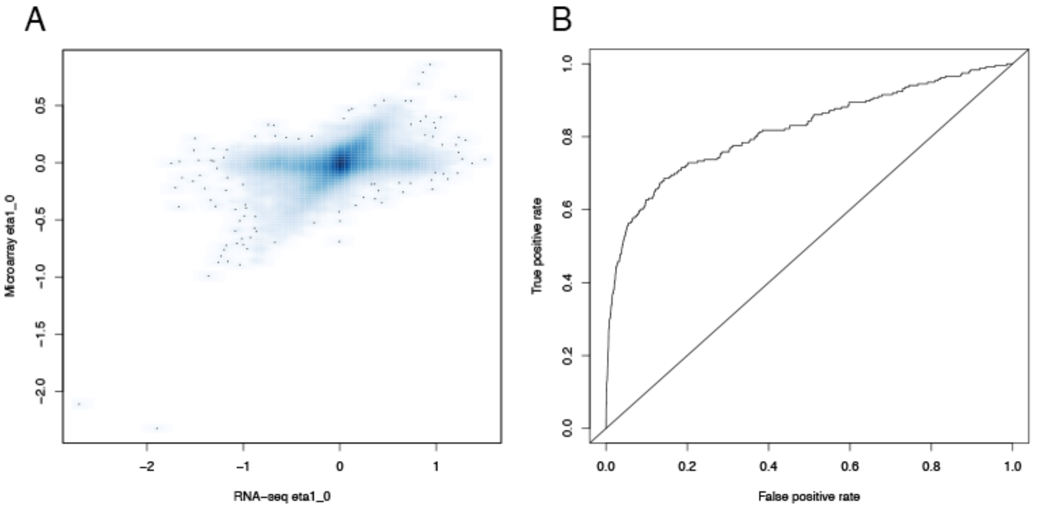

Supplementary Figure 1. Correlation of RNASeq and microarray data

(A) Scatterplot comparing eta1_0 (the calculated shift between polysomal and subpolysomal fractions) as derived from microarray and RNASeq data. The horizontal smear around microarray eta1_0=0 indicates numerous low-abundance mRNAs that are poorly resolved by microarray but which show larger changes in the RNASeq data.

(B) Receiver Operator Characteristic (ROC) curve of microarray vs RNASeq results thresholded at a posterior probability of 20% (taken as ‘ground truth’). The area under the curve is 72.5%.

Supplementary Figure 2

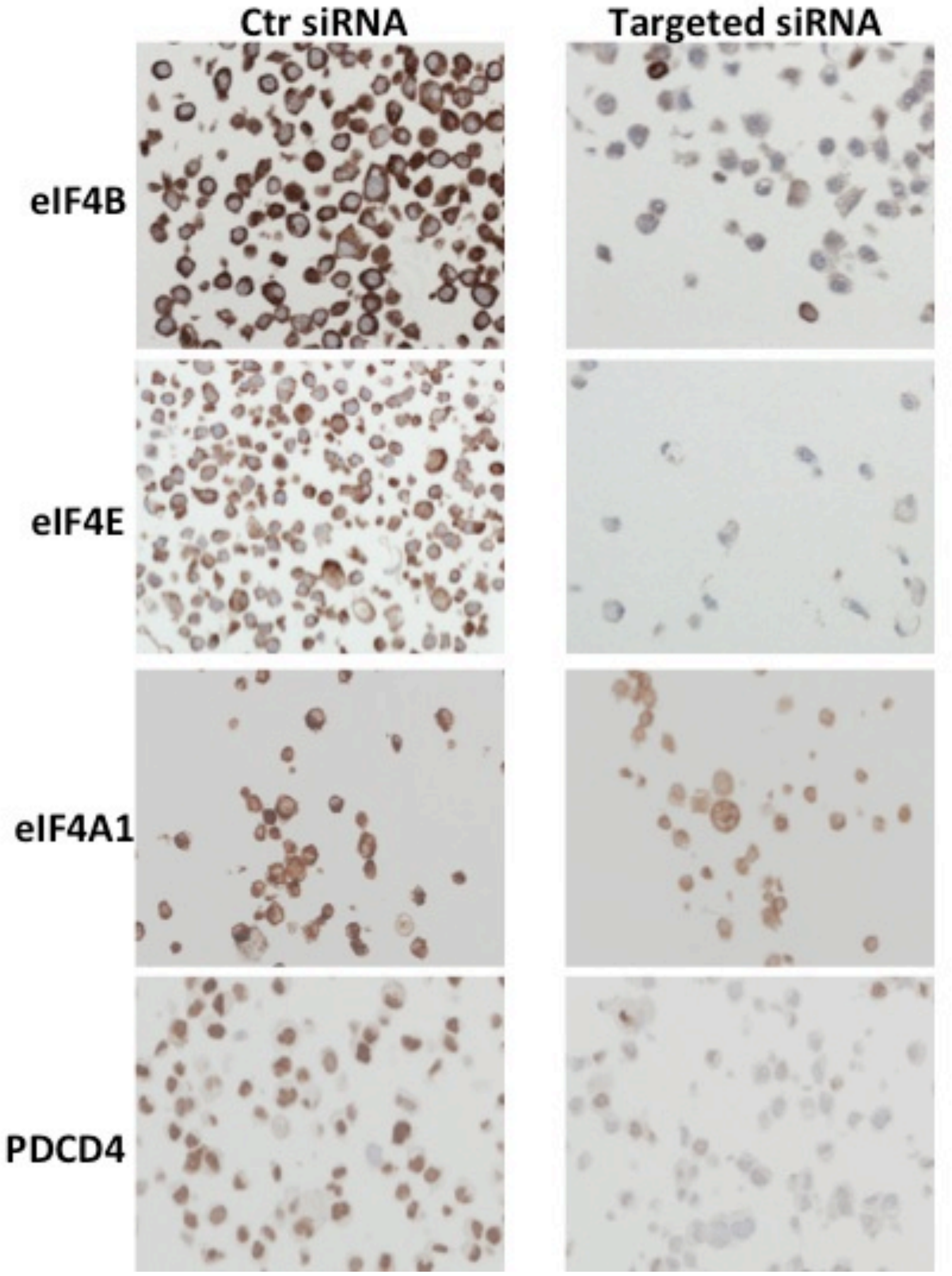

Supplementary Figure 2. Loss of immunohistochemical positivity in siRNA-treated cells demonstrates the specificity of the antibody assays. MCF7 cells were treated with respective siRNAs, collected and prepared as cytoblocks and immunohistochemical assays were performed.

Supplementary Figure 3.

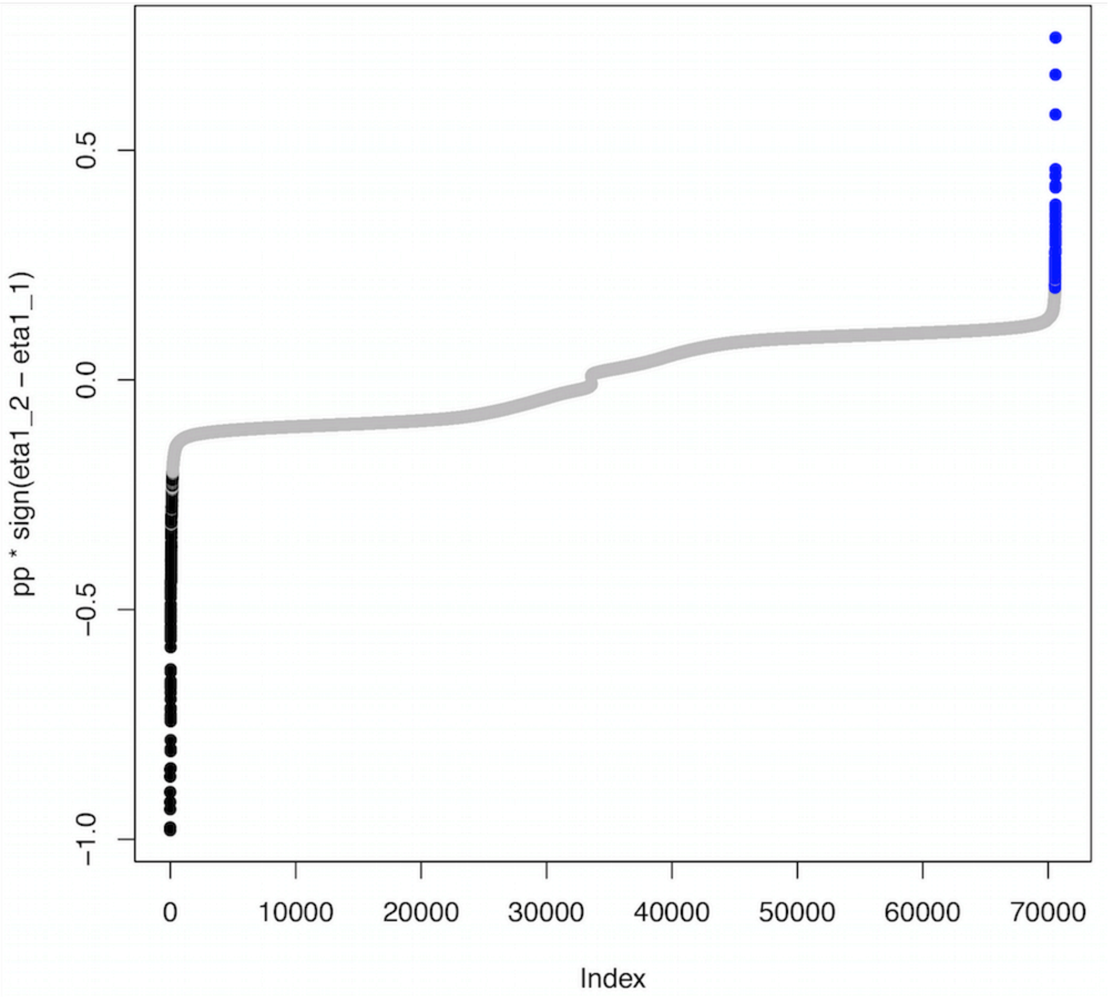

Supplementary Figure 3. Ranked list of eIF4A1-dependent and -independent mRNAs. eIF4A1-dependent messages are shown as black spots, and eIF4A1-independent mRNAs are in blue.
