# Supplementary material for: The malignant phenotype in breast cancer is driven by eIF4A1-mediated changes in the translational landscape
Source: Cell Death Dis. 2015 Jan 22;6(1):e1603–. doi: 10.1038/cddis.2014.542 (PMC4669741; doi:10.1038/cddis.2014.542)
Supplement: Supplementary Tables [file cddis2014542x2.pdf]

Supplementary Table 1. Summary of immunohistochemical data. Percentage proportions within each variable are shown in parentheses.

		eIF4E	eIF4A1	eIF4B	PDCD4(nuc)	PDCD4(cyt)
Intensity score	0 (Negative)	206(11)	243(11)	289(18)	783(28)	671(33)
	1 (Weak)	666(35)	928(40)	612(39)	1197(29)	689(51)
	2 (Moderate)	736(39)	914(39)	679(43)	337(27)	632(14)
	3 (Strong)	302(16)	236(10)	-	46(16)	371(2)
No of arrays		20	23	18	24	24
Total cores		3265	3724	2911	3903	3903
Missing data		1355	1421	1331	1540	1540

Supplementary Table 2. Univariate survival models. Variables reaching nominal significance (P<0.05) are shown in bold.

	ER negative disease					ER positive disease		
Variable	n	HR (95% CI)	P	T (95% CI)	P	n	HR (95% CI)	P
Grade	683	1.3 (1.0-1.7)	0.078	-	-	**2047**	**2.2 (1.8-2.8)**	**<0.001**
Nodes	**712**	**2.6 (2.1-3.2)**	**<0.001**	-	-	**2096**	**2.8 (2.4-3.3)**	**<0.001**
Size	**717**	**1.8 (1.2-2.3)**	**<0.001**	-	-	**2200**	**2.3 (1.8-2.8)**	**<0.001**
HER2	**651**	**1.5 1.1-2.2)**	**0.026**	-	-	**2038**	**2.3 (1.6-3.3)**	**<0.001**
AURORAK	529	1.0 (0.9-1.2)	0.736			**1576**	**1.6 (1.4-1.9)**	**<0.001**
eIF4E	**453**	**1.3 (1.0-1.6)**	**0.045**	-	-	1274	0.9 (0.8-1.1)	0.551
eIF4A1	**551**	**1.8 (1.1-3.0)**	**0.017**	**0.7 (0.5-1.0)**	**0.027**	1575	1.2 (0.6-2.4)	0.533
eIF4B	**394**	**1.5 (1.1-2.1)**	**0.008**	-	-	1037	1.0 (0.8-1.2)	0.841
PDCD4 (cyt)	558	0.9 (0.7-1.2)	0.392	-	-	**1584**	**0.6 (0.5-0.8)**	**<0.001**
PDCD4 (nuc)	558	1.0 (0.8-1.3)	0.756	-	-	**1584**	**0.6 (0.5-0.7)**	**<0.001**

Supplementary Table 3. Correlations between translation initiation factors and oestrogen receptor expression. Correlations with a significance <0.01 are shown in bold.

	eIF4E	eIF4A1	eIF4B	PDCD4 (cyt)	PDCD4 (nuc)	ER
eIF4E	-					
eIF4A1	**0.31**	-				
eIF4B	**0.33**	**0.34**	-			
PDCD4 (cyt)	**0.13**	**0.17**	**0.11**	-		
PDCD4 (nuc)	**0.10**	**0.13**	**0.13**	**0.41**	-	
ER	0.03	**-0.13**	0.06	**0.11**	**0.35**	-

Supplementary Table 4A. Associations between markers and clinicopathological data within the type of disease (ER-positive or ER-negative) in which the marker has prognostic value. Correlations reaching nominal significance (P<0.05) are shown in bold. Starred values refer to Fisher's exact test (cell values<5); all other P-values are chi-squared tests.

	ER Negative cases						ER Positive cases			
	eIF4E		eIF4A1		eIF4B		PDCD4(nuc)		PDCD4(cyt)	
	Low	High	Low	High	Low	High	Low	High	Low	High
Age at diagnosis										
≤55	263(71)	61(75)	35(81)	353(70)	178(78)	114(69)	215(64)	748(60)	288(60)	675(61)
>55	109(29)	20(25)	8(19)	155(31)	51(22)	51(31)	122(36)	499(40)	196(41)	425(39)
P-value	0.405		0.118*		0.053		0.203		0.485	
Tumour type										
Ductal	324(87)	69(85)	36(84)	443(87)	188(82)	141(86)	263(78)	911(73)	**341(71)**	**833(76)**
Lobular	13(4)	4(5)	4(9)	18(4)	12(5)	7(4)	47(14)	208(17)	**99(21)**	**156(14)**
Other	35(9)	8(10)	3(7)	46(9)	29(13)	17(10)	27(8)	127(10)	**44(9)**	**110(10)**
P-value	0.712*		0.175*		0.716*		0.182		**0.008**	
Grade										
1	19(6)	5(7)	**8(21)**	**18(4)**	**12(6)**	**5(3)**	**58(20)**	**282(25)**	102(24)	238(24)
2	82(24)	20(28)	**11(28)**	**115(25)**	**67(34)**	**30(20)**	**145(49)**	**632(56)**	228(54)	549(55)
3	241(71)	47(65)	**20(51)**	**319(71)**	**118(60)**	**115(77)**	**93(31)**	**220(19)**	95(22)	218(22)
P-value	0.652*		**<0.001***		**0.004***		**<0.001**		0.939	
Node Status										
Negative	190(55)	35(47)	20(54)	248(52)	109(51)	87(56)	**166(53)**	**730(64)**	259(59)	637(63)
Positive	154(45)	40(53)	17(46)	226(48)	106(49)	68(44)	**146(47)**	**411(36)**	183(41)	374(37)

P-value	0.178		0.839		0.302		**0.001**		0.112	
Tumour Size										
<20 mm	140(40)	27(35)	20(50)	198(42)	**94(44)**	**63(40)**	**157(49)**	**735(61)**	281(61)	611(58)
20-49 mm	198(56)	47(61)	19(48)	257(54)	**107(50)**	**90(58)**	**153(48)**	**437(36)**	168(36)	422(40)
≥50 mm	13(4)	3(4)	1(3)	21(4)	**15(7)**	**3(2)**	**10(3)**	**34(3)**	14(3)	30(3)
P-value	0.740*		0.641*		**0.044***		**0.001***		0.452	
HER2 Status										
Negative	275(81)	55(72)	35(85)	357(78)	170(79)	117(81)	286(88)	1055(91)	394(88)	947(91)
Positive	65(19)	21(28)	6(15)	103(22)	46(21)	27(19)	39(12)	102(9)	52(12)	89(9)
P-value	0.098		0.324*		0.556		0.084		0.065	
Aurora Kinase										
Negative	72(27)	13(20)	10(29)	79(21)	**47(28)**	**16(16)**	97(35)	295(31)	126(34)	266(31)
Positive	200(74)	51(80)	24(71)	295(79)	**124(73)**	**84(84)**	183(65)	649(69)	250(67)	582(69)
P-value	0.308		0.263		**0.031**		0.285		0.459	

Supplementary Table 4B. Remaining associations between markers and clinicopathological data (i.e. in the disease type lacking prognostic value for the variable).

	ER Positive cases						ER Negative cases			
	eIF4E		eIF4A1		eIF4B		PDCD4(nuc)		PDCD4(cyt)	
	Low	High	Low	High	Low	High	Low	High	Low	High
Age at diagnosis										
≤55	634(59)	130(65)	98(55)	860(62)	385(66)	295(65)	**187(68)**	**215(77)**	167(74)	235(71)
>55	439(41)	71(35)	79(45)	539(39)	201(34)	156(35)	**90(33)**	**66(24)**	59(26)	97(29)
P-value	0.138		0.117		0.923		**0.018**		0.422	
Tumour type										
Ductal	**777(73)**	**169(84)**	**120(68)**	**1054(75)**	**418(72)**	**341(76)**	239(86)	233(83)	195(86)	277(83)
Lobular	**188(18)**	**19(10)**	**42(24)**	**209(15)**	**121(21)**	**67(15)**	10(4)	20(7)	9(4)	21(6)
Other	**107(10)**	**13(7)**	**15(9)**	**135(10)**	**46(8)**	**42(9)**	28(10)	28(10)	22(10)	34(10)
P-value	**0.002**		**0.011**		**0.05**		0.195*		0.483*	
Grade										
1	**239(25)**	**36(20)**	**52(33)**	**276(22)**	129(25)	99(24)	15(6)	9(4)	11(6)	13(5)
2	**541(56)**	**88(49)**	**87(55)**	**699(55)**	287(55)	211(52)	64(25)	69(29)	51(25)	82(28)
3	**192(20)**	**54(30)**	**18(12)**	**294(23)**	103(20)	97(24)	173(69)	162(68)	139(69)	196(67)
P-value	**0.006**		**<0.001**		0.331		0.420*		0.723	
Node Status										
Negative	618(62)	111(61)	104(64)	797(62)	330(62)	255(61)	137(52)	150(58)	119(58)	168(53)
Positive	377(38)	71(39)	59(36)	495(38)	205(38)	163(39)	127(48)	109(42)	88(43)	148(47)
P-value	0.774		0.6		0.831		0.167		0.331	

Tumour Size										
<20 mm	593(58)	109(55)	**93(55)**	**792(59)**	**289(52)**	**271(63)**	**92(35)**	**127(48)**	91(44)	128(40)
20-49 mm	415(40)	81(41)	**75(45)**	**516(38)**	**256(46)**	**152(35)**	**153(59)**	**129(49)**	107(52)	175(55)
≥50 mm	24(2)	7(4)	**0(0)**	**42(3)**	**13(2)**	**9(2)**	**16(6)**	**7(3)**	9(4)	14(4)
P-value	0.507*		**0.013***		**0.002***		**0.004***		0.756*	
HER2 Status										
Negative	**957(93)**	**173(89)**	**161(98)**	**1194(90)**	513(92)	379(88)	186(75)	192(80)	**128(68)**	**250(84)**
Positive	**69(7)**	**21(11)**	**3(2)**	**135(10)**	47(8)	51(12)	62(25)	47(20)	**61(32)**	**48(16)**
P-value	**0.045**		**<0.001***		0.07		0.158		**<0.001**	
Aurora Kinase										
Negative	298(37)	41(29)	**77(54)**	**333(32)**	**179(40)**	**69(26)**	56(26)	39(19)	42(25)	53(21)
Positive	519(64)	102(71)	**65(46)**	**720(68)**	**268(60)**	**198(74)**	161(74)	166(81)	127(75)	200(79)
P-value	0.072		**<0.001**		**<0.001**		0.095		0.347	

Supplementary Table 5. eIF4A1-dependent and independent transcripts. eta1_1 is an estimate of the log fold change between subpolysomal fractions (siRNA vs ctrl) and eta1_2 is an estimate of the log fold change between polysomal fractions. Thresholding based on the posterior probability >0.2 was applied.

eIF4A1-dependent transcripts

No	Transcript ID	Gene name	Bayes factor	Posterior probability	eta1_1	eta1_2
1	ENST00000309340	MKNK2	430.88	0.98	0.74	-0.21
2	ENST00000264645	CASC3	338.08	0.97	1.07	0.26
3	ENST00000439930	PCBP2	128.08	0.93	0.63	-0.26
4	ENST00000546463	PCBP2	102.22	0.92	0.55	-0.28
5	ENST00000371085	GNAS	78.28	0.90	0.48	-0.15
6	ENST00000337979	STX3	56.83	0.86	0.57	-0.26
7	ENST00000373910	UBE2D1	49.91	0.85	-0.51	-1.58
8	ENST00000279101	CABLES2	49.02	0.84	0.00	-0.49
9	ENST00000439880	CDC25B	37.73	0.81	1.38	0.44
10	ENST00000265620	GNAS	36.82	0.80	0.54	-0.13
11	ENST00000350356	PRKACA	32.66	0.78	1.16	0.00
12	ENST00000250930	TMEM8A	26.05	0.74	0.51	-0.16
13	ENST00000237654	CCNI	25.28	0.74	0.63	-0.15
14	ENST00000308595	ADRBK1	25.18	0.74	1.02	0.23
15	ENST00000262891	MARK4	24.37	0.73	0.64	0.05
16	ENST00000318445	SLCO3A1	24.08	0.73	1.11	-0.02
17	ENST00000320580	PACS1	22.71	0.72	1.04	0.29
18	ENST00000242783	PKN1	22.44	0.71	0.88	0.03
19	ENST00000225394	GIT1	20.48	0.69	1.21	0.33

20	ENST00000377724	DCAF10	19.18	0.68	1.73	-0.22
21	ENST00000260600	ADCY3	18.20	0.67	1.00	0.12
22	ENST00000313578	AQP11	17.98	0.67	0.60	-0.26
23	ENST00000336395	TESK1	17.76	0.66	0.81	-0.02
24	ENST00000290649	AMFR	17.51	0.66	0.57	-0.13
25	ENST00000340833	CDC25B	17.40	0.66	1.72	0.45
26	ENST00000242209	FKBP9	17.12	0.66	0.55	0.00
27	ENST00000453954	TCF3	15.66	0.64	0.83	0.08
28	ENST00000323469	DIRAS1	15.27	0.63	0.38	-0.39
29	ENST00000323064	AGBL5	12.49	0.58	0.11	-0.51
30	ENST00000258530	APPL2	11.69	0.57	0.64	0.02
31	ENST00000374142	ZDHHC18	11.39	0.56	0.66	0.20
32	ENST00000415496	MGRN1	11.23	0.56	0.98	0.00
33	ENST00000339157	ABHD12	10.88	0.55	0.64	0.02
34	ENST00000261739	ANKRD13A	10.59	0.54	0.90	0.11
35	ENST00000415914	THAP5	10.41	0.54	-0.07	-0.79
36	ENST00000306090	GNAS	9.96	0.53	0.52	-0.14
37	ENST00000372591	FOXO6	9.88	0.52	0.98	0.13
38	ENST00000246548	UBA2	9.59	0.52	0.93	0.19
39	ENST00000389167	GPS2	9.49	0.51	1.09	0.26
40	ENST00000552819	PCBP2	9.15	0.50	0.47	-0.33
41	ENST00000367103	MAPKAPK2	9.05	0.50	0.78	0.17
42	ENST00000322030	SET	8.96	0.50	0.46	-0.12
43	ENST00000310496	RFNG	8.61	0.49	0.72	0.04
44	ENST00000319562	FARP1	8.10	0.47	0.33	-0.37
45	ENST00000376572	KIAA2013	7.76	0.46	0.52	-0.16
46	ENST00000381334	TMEM165	7.59	0.46	0.68	0.03
47	ENST00000258796	TTYH3	7.49	0.45	0.25	-0.47

48	ENST00000260403	TMEM62	7.47	0.45	0.88	0.17
49	ENST00000534680	C8orf82	7.16	0.44	0.46	-0.29
50	ENST00000422318	NT5DC2	7.09	0.44	0.35	-0.32
51	ENST00000313601	GNAI2	7.06	0.44	0.73	-0.14
52	ENST00000382998	CTIF	7.00	0.44	0.42	-0.88
53	ENST00000372991	CCND3	6.98	0.44	1.05	0.22
54	ENST00000355904	GTF2E2	6.91	0.43	0.68	0.02
55	ENST00000360938	ETS2	6.89	0.43	0.57	0.09
56	ENST00000221930	TGFB1	6.82	0.43	0.11	-0.57
57	ENST00000299564	CDYL2	6.68	0.43	0.23	-0.31
58	ENST00000361354	NCKAP1	6.60	0.42	0.15	-0.51
59	ENST00000379757	TXNDC5	6.35	0.41	0.03	-0.59
60	ENST00000325421	ZNF579	6.25	0.41	0.92	0.18
61	ENST00000262238	YY1	6.09	0.40	0.57	0.10
62	ENST00000454402	RFC5	6.05	0.40	0.58	-0.09
63	ENST00000366510	SCCPDH	5.88	0.40	0.35	-0.11
64	ENST00000460006	CDS2	5.88	0.40	-0.37	-1.11
65	ENST00000379380	LYRM7	5.87	0.39	-0.08	-0.81
66	ENST00000506817	RHOBTB3	5.82	0.39	0.77	-0.48
67	ENST00000553630	CALM1	5.78	0.39	0.37	-0.14
68	ENST00000354359	GNAS	5.75	0.39	0.61	-0.14
69	ENST00000276282	MFHAS1	5.74	0.39	0.70	0.29
70	ENST00000378103	GBA2	5.74	0.39	0.43	-0.09
71	ENST00000233154	NCK2	5.57	0.38	0.50	-0.20
72	ENST00000355415	MFSD12	5.47	0.38	0.78	-0.09
73	ENST00000329286	RAB12	5.32	0.37	0.61	0.06
74	ENST00000376834	C9orf41	5.31	0.37	-0.15	-0.85
75	ENST00000352397	CYB5R3	5.15	0.36	0.65	-0.01

76	ENST00000358173	EPHB4	5.15	0.36	0.31	-0.22
77	ENST00000291900	ZER1	5.14	0.36	0.61	0.13
78	ENST00000222308	FKBP8	5.08	0.36	0.64	-0.09
79	ENST00000298585	ZMYND19	5.03	0.36	0.76	0.30
80	ENST00000396684	ATN1	4.94	0.35	0.87	0.09
81	ENST00000361434	LPHN1	4.70	0.34	0.48	-0.15
82	ENST00000373218	EIF4EBP2	4.67	0.34	-0.05	-0.66
83	ENST00000263025	MAPK3	4.52	0.33	0.49	-0.21
84	ENST00000431526	ZNF598	4.44	0.33	1.06	0.23
85	ENST00000409140	SPATS2L	4.43	0.33	0.79	0.04
86	ENST00000234111	ODC1	4.33	0.32	0.30	-0.38
87	ENST00000228495	KCTD10	4.13	0.31	-0.10	-0.60
88	ENST00000250916	KLF16	4.05	0.31	0.85	0.28
89	ENST00000078429	GNA11	4.02	0.31	0.79	0.19
90	ENST00000369109	APH1A	4.00	0.31	0.16	-0.22
91	ENST00000513339	REEP5	3.91	0.30	1.25	-0.70
92	ENST00000395423	TCF3	3.86	0.30	0.81	0.05
93	ENST00000275766	ZC3HAV1L	3.84	0.30	0.30	-0.22
94	ENST00000557441	CCNK	3.79	0.30	0.81	-0.34
95	ENST00000433375	GLIS2	3.76	0.29	0.61	0.08
96	ENST00000341446	BRI3BP	3.75	0.29	0.42	-0.06
97	ENST00000380243	CCDC85C	3.68	0.29	0.88	0.13
98	ENST00000289703	ELK4	3.64	0.29	0.75	-0.58
99	ENST00000306666	PTGER3	3.62	0.29	-0.14	-1.05
100	ENST00000402868	SETD8	3.60	0.29	0.39	-0.18
101	ENST00000406655	PPP2CB	3.60	0.29	0.82	0.20
102	ENST00000339438	CSDE1	3.60	0.29	0.52	-0.08
103	ENST00000368954	CERS2	3.56	0.28	0.44	-0.11

104	ENST00000368594	INPP5A	3.52	0.28	0.21	-0.20
105	ENST00000525817	NPEPL1	3.50	0.28	0.78	-0.17
106	ENST00000320619	ZNRF1	3.47	0.28	0.29	-0.39
107	ENST00000219794	BCKDK	3.46	0.28	0.35	-0.41
108	ENST00000431765	DVL3	3.46	0.28	1.25	-0.12
109	ENST00000374270	SUSD1	3.45	0.28	0.62	-0.31
110	ENST00000541272	UBC	3.44	0.28	0.67	-0.78
111	ENST00000058691	HEBP2	3.43	0.28	0.51	0.03
112	ENST00000204566	SPG21	3.43	0.28	0.49	-0.13
113	ENST00000301732	ABCA3	3.43	0.28	0.61	0.18
114	ENST00000359282	PCBP2	3.41	0.28	0.34	-0.42
115	ENST00000326840	DCBLD2	3.41	0.27	0.23	-0.38
116	ENST00000405675	CSNK1E	3.41	0.27	0.52	0.01
117	ENST00000468435	UHRF2	3.40	0.27	1.08	-0.67
118	ENST00000275364	GNA12	3.39	0.27	0.04	-0.32
119	ENST00000376372	WDR45	3.37	0.27	0.00	-0.58
120	ENST00000250896	MKNK2	3.33	0.27	0.36	-0.28
121	ENST00000375107	AGPAT1	3.26	0.27	0.68	0.11
122	ENST00000374557	EPB41L4B	3.23	0.26	0.97	0.29
123	ENST00000302277	ZNF804A	3.22	0.26	0.55	-0.56
124	ENST00000357324	ATP13A1	3.21	0.26	0.57	-0.28
125	ENST00000375793	PLEKHM2	3.21	0.26	0.83	0.27
126	ENST00000434316	TMCO3	3.18	0.26	0.12	-0.35
127	ENST00000309909	FBXW8	3.16	0.26	0.53	-0.51
128	ENST00000534699	CSDE1	3.16	0.26	0.43	-0.24
129	ENST00000415050	C14orf109	3.14	0.26	0.55	-0.05
130	ENST00000324093	PLXND1	3.09	0.26	0.20	-0.32
131	ENST00000359280	ADAM15	3.07	0.25	1.47	-0.04

132	ENST00000355849	KHDRBS3	3.01	0.25	1.25	0.26
133	ENST00000219548	STUB1	3.00	0.25	0.48	0.02
134	ENST00000366624	RP5-862P8.2	2.99	0.25	0.44	-0.22
135	ENST00000525266	ZNF7	2.98	0.25	0.87	-0.18
136	ENST00000409939	TBC1D10B	2.95	0.25	0.77	0.18
137	ENST00000367142	NUCKS1	2.93	0.25	0.62	-0.02
138	ENST00000359246	PHF2	2.89	0.24	0.50	0.12
139	ENST00000373896	PHF19	2.87	0.24	0.39	0.08
140	ENST00000546652	PCBP2	2.86	0.24	0.69	-0.17
141	ENST00000405333	ODC1	2.84	0.24	0.45	-0.17
142	ENST00000281187	VPS26B	2.82	0.24	0.60	0.21
143	ENST00000377045	ARAF	2.81	0.24	0.46	-0.01
144	ENST00000335255	FOXK2	2.80	0.24	0.53	0.07
145	ENST00000457361	IKZF2	2.78	0.24	0.22	-0.37
146	ENST00000379016	HIPK3	2.68	0.23	0.16	-0.51
147	ENST00000391831	AKT1S1	2.67	0.23	0.27	-1.27
148	ENST00000355300	LINGO1	2.66	0.23	0.63	-0.17
149	ENST00000541297	CLPTM1	2.61	0.22	1.44	-0.06
150	ENST00000503731	TUSC3	2.59	0.22	0.51	-0.22
151	ENST00000513317	ACSL1	2.56	0.22	0.50	-0.53
152	ENST00000504639	PAIP1	2.54	0.22	0.84	-0.36
153	ENST00000458427	JRKL	2.50	0.22	0.37	-0.63
154	ENST00000228251	CSDA	2.49	0.22	0.57	0.10
155	ENST00000549863	PCBP2	2.48	0.22	0.78	-0.02
156	ENST00000246163	MAX	2.47	0.22	1.24	0.33
157	ENST00000361246	CDC42BPB	2.47	0.22	0.77	0.35
158	ENST00000276033	SLC16A2	2.45	0.21	0.67	0.14

159	ENST00000547026	ZNF641	2.44	0.21	0.55	-0.55
160	ENST00000328965	OAF	2.44	0.21	0.53	-0.03
161	ENST00000263121	SMARCB1	2.42	0.21	0.96	0.23
162	ENST00000262325	AP2B1	2.41	0.21	1.28	0.44
163	ENST00000327490	FAM100B	2.40	0.21	0.46	-0.12
164	ENST00000323267	TMEM63B	2.38	0.21	-0.05	-0.43
165	ENST00000547573	C12orf76	2.38	0.21	0.47	-0.68
166	ENST00000396247	RDH13	2.36	0.21	0.31	-0.93
167	ENST00000262160	SMAD2	2.34	0.21	0.23	-0.38
168	ENST00000369446	F8A1	2.32	0.20	0.80	0.33
169	ENST00000535361	STX3	2.32	0.20	0.64	-0.32
170	ENST00000513588	CTNND2	2.31	0.20	1.12	0.34
171	ENST00000007390	TSR3	2.31	0.20	0.61	0.07
172	ENST00000456764	IGFBP2	2.30	0.20	0.36	-0.42
173	ENST00000339526	GLUL	2.30	0.20	0.38	-0.07
174	ENST00000248996	GNAZ	2.29	0.20	0.71	0.20
175	ENST00000461735	SLC4A2	2.26	0.20	0.83	-0.63

eIF4A1-independent transcripts

No	Transcript ID	Gene name	Bayes factor	Posterior probability	eta1_1	eta1_2
1	ENST00000263233	SYP	26.40	0.75	0.09	1.58
2	ENST00000270066	SMG9	17.84	0.66	-0.16	0.33
3	ENST00000320270	RRS1	12.31	0.58	-0.01	0.51
4	ENST00000306014	DDX54	7.65	0.46	-0.78	0.31
5	ENST00000300540	ZNF85	7.19	0.44	-0.48	0.65
6	ENST00000474861	HBXIP	6.64	0.42	-0.96	0.88

7	ENST00000311922	TRIB1	6.53	0.42	-0.15	0.35
8	ENST00000246229	PLAGL2	6.51	0.42	-0.11	0.27
9	ENST00000336139	ZNF202	5.56	0.38	-0.98	0.52
10	ENST00000456458	NT5C3	5.46	0.38	-0.24	0.59
11	ENST00000351986	NPM1	5.33	0.37	-0.48	0.08
12	ENST00000354241	TRIM4	5.09	0.36	-0.46	0.09
13	ENST00000253024	TRIM28	4.98	0.36	-0.33	0.27
14	ENST00000340913	HNRNPA1	4.76	0.35	-0.68	-0.06
15	ENST00000546591	RPL41	4.51	0.33	-0.17	0.41
16	ENST00000373078	NAIF1	4.29	0.32	-0.57	0.03
17	ENST00000378643	FANCG	4.17	0.32	-0.47	0.11
18	ENST00000526453	RP11-110I1.12	4.02	0.31	-0.79	0.21
19	ENST00000216714	APEX1	3.94	0.30	-0.54	0.00
20	ENST00000527791	RPS25	3.85	0.30	-0.09	0.68
21	ENST00000394287	MED1	3.80	0.30	-0.37	0.27
22	ENST00000524496	RPL27A	3.55	0.28	-0.15	0.38
23	ENST00000506763	HAUS3	3.47	0.28	-0.29	0.68
24	ENST00000396113	NDUFAF6	3.45	0.28	-0.77	0.50
25	ENST00000369722	ATP5F1	3.44	0.28	-0.61	0.32
26	ENST00000431643	EIF3B	3.26	0.27	-0.69	1.65
27	ENST00000462426	DYNC2LI1	3.23	0.26	-0.94	0.66
28	ENST00000239165	HOXB7	3.11	0.26	-0.16	0.44
29	ENST00000309668	INHBC	3.07	0.25	-1.38	0.59
30	ENST00000355057	HIST1H4J	3.04	0.25	-0.34	0.45
31	ENST00000344683	MCPH1	2.97	0.25	-0.88	0.09
32	ENST00000335987	OVOL1	2.88	0.24	-0.37	0.02
33	ENST00000367166	TFB1M	2.82	0.24	-0.34	0.29

34	ENST00000238667	FLVCR2	2.79	0.24	-0.67	-0.29
35	ENST00000394800	MATR3	2.71	0.23	-0.62	1.11
36	ENST00000328550	ZNF571	2.69	0.23	-1.05	0.80
37	ENST00000409468	HSPE1	2.66	0.23	0.04	0.79
38	ENST00000551132	CNOT2	2.64	0.23	-0.65	0.50
39	ENST00000330368	ACTRT3	2.62	0.23	-0.41	0.28
40	ENST00000335117	ZNF486	2.57	0.22	-0.42	0.33
41	ENST00000380290	FAM96A	2.54	0.22	-0.15	0.49
42	ENST00000534176	UBXN1	2.43	0.21	-0.31	0.26
43	ENST00000318317	LRR1	2.41	0.21	-0.29	0.55
44	ENST00000373727	MRRF	2.38	0.21	-0.65	0.64
45	ENST00000254166	ZNF132	2.35	0.21	-0.78	0.22
46	ENST00000221431	FBXO17	2.34	0.21	-0.35	0.15
47	ENST00000257979	MIP	2.31	0.20	-0.39	0.71
48	ENST00000261070	COX17	2.31	0.20	-0.04	0.35
49	ENST00000360441	HIST1H4E	2.27	0.20	-0.65	0.27

Supplementary Table 6. Analysis of 5’ UTR characteristics of translationally eIF4A1-dependent and independent mRNAs. The Kolmogorov-Smirnov test was performed for the parameters listed for eIF4A1-dependent and eIF4A1-independent transcript. D= Kolmogorov–Smirnov statistic.

**5'UTR characterisics**	**D**	**p-value**
GC content	0.59	6.20E-12
C content	0.45	4.30E-07
G content	0.44	1.20E-06
ΔG last 50nt	0.40	1.20E-05
ΔG full length	0.29	3.60E-03
ΔG first 50nt	0.28	4.90E-03
ΔG first 1000nt	0.24	2.90E-02
length	0.22	5.10E-02
AUG count	0.16	2.90E-01

Supplementary Table 8. Details of immunohistochemical assays.

Antigen	Antibody	Supplier	Antigen retrieval method	Antibody concentration	Incubation conditions	Notes
eIF4E	sc-9976 (mouse mono)	Santa Cruz	Citrate 20' at 100C	1:100	15' at room temp	
eIF4A1	ab31217 (rabbit poly)	Abcam	edta 20' at 100C	1:50	60' at room temp	antibody diluted in 300mM TBS with serum block to reduce background.
eIF4B	2232-1 (rabbit mono)	Epitomics	edta 20' at 100C	1:100	15' at room temp	
PDCD4	ab80590 (rabbit mono)	Abcam	edta 20' at 100C	1:1500	15' at room temp	

Supplementary Table 9. Numbers at risk in Kaplan-Meier analyses.

Time (Years)		Number at risk 0	2	4	6	8	10
eIF4E	0	0	42	52	49	45	35
	1	0	104	119	118	103	86
	2	0	110	113	103	86	69
	3	0	57	51	53	48	39

Time (Years)		Number at risk 0	2	4	6	8	10
eIF4A1	0	0	29	38	37	35	28
	1	0	128	136	124	124	106
	2	0	154	157	128	128	98
	3	0	68	75	54	54	36

Time (Years)		Number at risk 0	2	4	6	8	10
eIF4B	0	0	56	59	60	58	50
	1	0	109	118	114	109	93
	2	0	100	111	99	85	65

Time (Years)		Number at risk 0	2	4	6	8	10
PDCD4 (nuc)	0	0	221	269	268	230	159
	1	0	317	389	380	312	217
	2	1	354	421	413	358	269
	3	0	211	250	232	194	146
