# Supplementary material for: The malignant phenotype in breast cancer is driven by eIF4A1-mediated changes in the translational landscape
Source: Cell Death Dis. 2015 Jan 22;6(1):e1603–. doi: 10.1038/cddis.2014.542 (PMC4669741; doi:10.1038/cddis.2014.542)
Supplement: Supplementary Tables [file cddis2014542x3.pdf]

Supplementary Table 7. Over-representation and Gene Set Enrichment analyses of eIF4A1-dependence at the level of translation (polysome/subpolysome redistribution) and total mRNA abundance.

Over-representation analysis (protein families)
All non-redundant categories with >2 genes shown

**eIF4A1-dependent mRNAs**	**P (FDR)**	**Expected no**	**Observed no**	**Genes**
G-protein alpha subunit	3.93E-06	0.130685	5	GNAZ GNAI2 GNA11 GNAS GNA12
Cyclin, N-terminal domain	0.00313468	0.296218	4	CCNI CCND3 CCNK CABLES2
Protein kinase domain	0.00316199	3.13643	11	ADRBK1 MARK4 TESK1 CDC42BPB CSNK1E PRKACA PKN1 HIPK3 MKNK2 MAPKAPK2 MAPK3
**eIF4A1-independent mRNAs**				
Zinc finger, C2H2 type	0.0102672	1.40973	6	6 ZNF571 ZNF132 OVOL1 PLAGL2 ZNF85 ZNF202

GSEA Analysis (eIF4A1-dependence)
Top 20 categories with FDR<0.25 shown
eIF4A1-dependent gene sets

	**SIZE**	**ES**	**NES**	**NOM p-val**	**FDR q-val**	**FWER p-val**	**RANK AT MAX**	**EADING EDGE**
**Biocarta categories**								
BIOCARTA_CREB_PATHWAY	27	-0.54233336	-2.5955534	0	0.002617879	0.002	3050	tags=52%, list=16%, signal=61%
BIOCARTA_ERK_PATHWAY	28	-0.5300096	-2.4911392	0	0.004615376	0.007	955	tags=25%, list=5%, signal=26%
BIOCARTA_GPCR_PATHWAY	34	-0.4527145	-2.3306682	0	0.012513909	0.027	854	tags=24%, list=4%, signal=25%
BIOCARTA_MAPK_PATHWAY	85	-0.32819548	-2.2327616	0	0.021895325	0.064	1933	tags=25%, list=10%, signal=27%
BIOCARTA_BARRESTIN_SRC_PATHWAY	15	-0.56624985	-2.1756601	0	0.029599514	0.105	235	tags=20%, list=1%, signal=20%
BIOCARTA_IGF1R_PATHWAY	23	-0.46399823	-2.1142561	0.003738318	0.040106993	0.163	3050	tags=48%, list=16%, signal=57%
BIOCARTA_BAD_PATHWAY	26	-0.45122012	-2.0612748	0.005825243	0.04141674	0.239	3264	tags=50%, list=17%, signal=60%
BIOCARTA_CHREBP2_PATHWAY	42	-0.37067717	-2.0760975	0.005671077	0.042666525	0.223	3493	tags=40%, list=18%, signal=49%
BIOCARTA_GCR_PATHWAY	19	-0.48994818	-2.0832496	0.001996008	0.04606622	0.213	1993	tags=37%, list=10%, signal=41%
BIOCARTA_TGFB_PATHWAY	19	-0.458955	-1.9603087	0.003992016	0.07135666	0.411	2012	tags=32%, list=10%, signal=35%
BIOCARTA_AKAPCENTROSOME_PATHWAY	15	-0.51586366	-1.9439404	0.007797271	0.07307961	0.444	3050	tags=47%, list=16%, signal=55%
BIOCARTA_TEL_PATHWAY	18	-0.45313245	-1.8903471	0.009881423	0.0938439	0.565	2866	tags=50%, list=15%, signal=59%
BIOCARTA_FMLP_PATHWAY	34	-0.34672955	-1.7761961	0.019120459	0.13744554	0.804	3536	tags=38%, list=18%, signal=47%
BIOCARTA_MPR_PATHWAY	34	-0.35035113	-1.7980186	0.021526419	0.13755181	0.768	886	tags=18%, list=5%, signal=18%
BIOCARTA_PYK2_PATHWAY	27	-0.3700757	-1.7816187	0.015209125	0.1412102	0.797	3094	tags=37%, list=16%, signal=44%
BIOCARTA_GSK3_PATHWAY	27	-0.37915888	-1.8046193	0.018621974	0.14248395	0.751	5497	tags=59%, list=28%, signal=83%
BIOCARTA_CCR3_PATHWAY	23	-0.40172747	-1.756542	0.017142856	0.14360377	0.835	235	tags=13%, list=1%, signal=13%
BIOCARTA_IL2RB_PATHWAY	38	-0.33062506	-1.7341974	0.021400778	0.14572135	0.866	2933	tags=37%, list=15%, signal=43%
BIOCARTA_CSK_PATHWAY	22	-0.38944396	-1.7404138	0.025145067	0.14850758	0.856	424	tags=14%, list=2%, signal=14%
BIOCARTA_VIP_PATHWAY	25	-0.37137872	-1.6913317	0.029940119	0.1705908	0.912	1283	tags=32%, list=7%, signal=34%
**Reactome categories**								
REACTOME_DAG_AND_IP3_SIGNALING	31	-0.62402356	-3.1233697	0	0	0	1377	tags=39%, list=7%, signal=42%
REACTOME_PLC_BETA_MEDIATED_EVENTS	42	-0.56943005	-3.117028	0	0	0	1377	tags=33%, list=7%, signal=36%
REACTOME_CA_DEPENDENT_EVENTS	29	-0.63909024	-3.1079552	0	0	0	1377	tags=41%, list=7%, signal=44%
REACTOME_DOWNSTREAM_SIGNAL_TRANSDUCTION	91	-0.4261822	-2.9814339	0	0	0	3050	tags=36%, list=16%, signal=43%
REACTOME_OPIOID_SIGNALLING	76	-0.44493353	-2.9120543	0	0	0	1377	tags=26%, list=7%, signal=28%
REACTOME_SIGNALING_BY_FGFR	107	-0.39662504	-2.8903675	0	0	0	2092	tags=29%, list=11%, signal=32%
REACTOME_NGF_SIGNALLING_VIA_TRKA_FROM_THE_PLASMA_MEMBRANE	133	-0.37264517	-2.884793	0	0	0	2112	tags=29%, list=11%, signal=32%
REACTOME_GLUCAGON_SIGNALING_IN_METABOLIC_REGULATION	32	-0.56314605	-2.817463	0	0	0	790	tags=25%, list=4%, signal=26%
REACTOME_SIGNALLING_BY_NGF	211	-0.32020423	-2.7881958	0	0	0	2112	tags=27%, list=11%, signal=30%
REACTOME_SIGNALING_BY_ERBB2	97	-0.39203283	-2.7732968	0	0	0	2092	tags=28%, list=11%, signal=31%
REACTOME_SIGNALING_BY_FGFR_IN_DISEASE	121	-0.36532968	-2.7553017	0	0	0	2092	tags=27%, list=11%, signal=30%
REACTOME_DOWNSTREAM_SIGNALING_OF_ACTIVATED_FGFR	96	-0.3818161	-2.737539	0	0	0	2092	tags=27%, list=11%, signal=30%
REACTOME_SIGNALING_BY_EGFR_IN_CANCER	105	-0.37714633	-2.730975	0	0	0	2112	tags=27%, list=11%, signal=30%
REACTOME_PHOSPHOLIPASE_C_MEDIATED_CASCADE	52	-0.46923918	-2.684026	0	6.96E-05	0.001	1377	tags=27%, list=7%, signal=29%
REACTOME_REGULATION_OF_WATER_BALANCE_BY_RENAL_AQUAPORINS	42	-0.48701996	-2.702312	0	7.43E-05	0.001	1377	tags=24%, list=7%, signal=26%
REACTOME_SIGNALING_BY_PDGF	118	-0.36435795	-2.7103796	0	7.96E-05	0.001	3050	tags=32%, list=16%, signal=38%
REACTOME_AQUAPORIN_MEDIATED_TRANSPORT	49	-0.4354774	-2.4930024	0	4.54E-04	0.007	1377	tags=22%, list=7%, signal=24%
REACTOME_DARPP_32_EVENTS	24	-0.55811363	-2.5055034	0	4.81E-04	0.007	957	tags=38%, list=5%, signal=39%
REACTOME_PKA_MEDIATED_PHOSPHORYLATION_OF_CREB	17	-0.6006834	-2.3846416	0.001926782	0.001540262	0.024	1377	tags=35%, list=7%, signal=38%
REACTOME_INTEGRATION_OF_ENERGY_METABOLISM	115	-0.32096735	-2.3771482	0	0.001578756	0.026	1410	tags=17%, list=7%, signal=19%
**GO categories**								
REGULATION_OF_PROTEIN_MODIFICATION_PROCESS	44	-0.3937945	-2.2585683	0	0.048124332	0.202	2750	tags=32%, list=14%, signal=37%
PROTEIN_AMINO_ACID_PHOSPHORYLATION	271	-0.24972406	-2.2691388	0	0.054979853	0.187	2750	tags=25%, list=14%, signal=29%
IMMUNE_EFFECTOR_PROCESS	38	-0.42197832	-2.2183442	0.003584229	0.05769448	0.283	5283	tags=63%, list=27%, signal=87%
POST_TRANSLATIONAL_PROTEIN_MODIFICATION	468	-0.2272993	-2.3314106	0	0.06028459	0.11	3262	tags=25%, list=17%, signal=30%
OXIDOREDUCTASE_ACTIVITY_ACTING_ON_NADH_OR_NADPH	25	-0.48549327	-2.27604	0	0.068132125	0.176	2890	tags=52%, list=15%, signal=61%
PHOSPHORYLATION	304	-0.22965401	-2.1268992	0	0.083290465	0.522	2553	tags=22%, list=13%, signal=25%
PROTEIN_UBIQUITINATION	40	-0.3902988	-2.137502	0	0.08749176	0.499	509	tags=15%, list=3%, signal=15%
TUBULIN_BINDING	47	-0.35568342	-2.0217686	0.005617978	0.088345	0.792	2918	tags=34%, list=15%, signal=40%
PROTEIN_KINASE_ACTIVITY	279	-0.22321065	-2.0458796	0	0.091209635	0.737	3327	tags=27%, list=17%, signal=32%
UBIQUITIN_CYCLE	48	-0.3441603	-2.024517	0.001855288	0.09154453	0.784	509	tags=13%, list=3%, signal=13%
COVALENT_CHROMATIN_MODIFICATION	25	-0.45949483	-2.0974743	0	0.09185648	0.594	1133	tags=36%, list=6%, signal=38%
PROTEIN_MODIFICATION_PROCESS	623	-0.19204713	-2.031397	0	0.0937083	0.772	2226	tags=18%, list=11%, signal=19%
PROTEIN_MODIFICATION_BY_SMALL_PROTEIN_CONJUGATION	43	-0.3799313	-2.0726411	0	0.09429154	0.669	694	tags=16%, list=4%, signal=17%
HISTONE_MODIFICATION	24	-0.4388304	-2.0608406	0.003824092	0.09498083	0.701	1133	tags=33%, list=6%, signal=35%
PHOSPHOTRANSFERASE_ACTIVITY_ALCOHOL_GROUP_AS_ACCEPTOR	326	-0.22289748	-2.1449869	0	0.0951585	0.479	3927	tags=31%, list=20%, signal=38%
PROTEIN_KINASE_CASCADE	289	-0.21705455	-2.0026777	0	0.09567664	0.834	2570	tags=23%, list=13%, signal=26%
BIOPOLYMER_MODIFICATION	642	-0.19192052	-2.0470195	0	0.09699101	0.735	2226	tags=18%, list=11%, signal=20%
POSITIVE_REGULATION_OF_PROTEIN_MODIFICATION_PROCESS	29	-0.4252713	-2.0740428	0.001976285	0.10136102	0.664	589	tags=17%, list=3%, signal=18%
**KEGG categories**								
KEGG_COLORECTAL_CANCER	62	-0.4091385	-2.5179148	0	0.001192593	0.003	4174	tags=50%, list=22%, signal=64%
KEGG_LONG_TERM_DEPRESSION	70	-0.3834733	-2.4866211	0	0.001500783	0.005	1509	tags=24%, list=8%, signal=26%
KEGG_MELANOGENESIS	101	-0.3432427	-2.5228963	0	0.001788889	0.003	854	tags=13%, list=4%, signal=13%
KEGG_ENDOMETRIAL_CANCER	52	-0.41995326	-2.4494536	0	0.002110235	0.009	4174	tags=54%, list=22%, signal=68%
KEGG_PROGESTERONE_MEDIATED_OOCYTE_MATURATION	84	-0.37418714	-2.5450108	0	0.003577778	0.003	1657	tags=24%, list=9%, signal=26%
KEGG_GNRH_SIGNALING_PATHWAY	100	-0.3230261	-2.3402925	0	0.003841127	0.02	3501	tags=30%, list=18%, signal=36%
KEGG_PHOSPHATIDYLINOSITOL_SIGNALING_SYSTEM	76	-0.34461805	-2.2825053	0	0.004468318	0.027	2917	tags=38%, list=15%, signal=45%
KEGG_OOCYTE_MEIOSIS	109	-0.31234506	-2.2765865	0	0.004614209	0.032	3118	tags=30%, list=16%, signal=36%
KEGG_GAP_JUNCTION	88	-0.32101044	-2.213696	0	0.005896904	0.055	830	tags=13%, list=4%, signal=13%
KEGG_INSULIN_SIGNALING_PATHWAY	136	-0.2824003	-2.206712	0	0.006116179	0.061	3130	tags=27%, list=16%, signal=32%
KEGG_VASOPRESSIN_REGULATED_WATER_REABSORPTION	44	-0.38561144	-2.2167768	0	0.006486594	0.055	185	tags=9%, list=1%, signal=9%
KEGG_DILATED_CARDIOMYOPATHY	89	-0.32169828	-2.226943	0	0.00693689	0.053	4308	tags=37%, list=22%, signal=47%
KEGG_VIBRIO_CHOLERAE_INFECTION	54	-0.36529863	-2.147904	0.001897533	0.008627246	0.089	536	tags=11%, list=3%, signal=11%
KEGG_LONG_TERM_POTENTIATION	69	-0.32365182	-2.0991592	0.001766785	0.010486206	0.119	3291	tags=32%, list=17%, signal=38%
KEGG_VASCULAR_SMOOTH_MUSCLE_CONTRACTION	115	-0.28012586	-2.0744958	0.001730104	0.011968696	0.144	287	tags=9%, list=1%, signal=9%
KEGG_WNT_SIGNALING_PATHWAY	149	-0.255164	-2.0207522	0	0.017261386	0.21	3379	tags=29%, list=17%, signal=35%
KEGG_MAPK_SIGNALING_PATHWAY	260	-0.21783839	-1.9876659	0	0.021213107	0.27	2126	tags=19%, list=11%, signal=21%
KEGG_NEUROTROPHIN_SIGNALING_PATHWAY	123	-0.25646883	-1.9216363	0	0.03143508	0.384	2933	tags=30%, list=15%, signal=35%
KEGG_RENAL_CELL_CARCINOMA	70	-0.29776672	-1.9130478	0.001754386	0.031674974	0.405	3940	tags=34%, list=20%, signal=43%
KEGG_GLIOMA	64	-0.30039573	-1.8961023	0.005309735	0.0330815	0.435	2933	tags=31%, list=15%, signal=37%

eIF4A1-independent gene sets

	**SIZE**	**ES**	**NES**	**NOM p-val**	**FDR q-val**	**FWER p-val**	**RANK AT MAX**	**EADING EDGE**
**Biocarta pathways**								
BIOCARTA_ATM_PATHWAY	20	0.5128766	2.3067076	0	0.014876452	0.025	3765	tags=65%, list=19%, signal=81%
**Reactome categories**								
REACTOME_PEPTIDE_CHAIN_ELONGATION	84	0.30287075	2.2139184	0	0.09512686	0.112	9587	tags=80%, list=49%, signal=157%
REACTOME_INFLUENZA_VIRAL_RNA_TRANSCRIPTION_AND_REPLICATION	100	0.268495	2.0212114	0	0.10381648	0.404	9587	tags=75%, list=49%, signal=148%
REACTOME_SRP_DEPENDENT_COTRANSLATIONAL_PROTEIN_TARGETING_TO_MEMBRANE	107	0.26355752	2.04156	0.002277904	0.11838357	0.361	9587	tags=76%, list=49%, signal=149%
REACTOME_3_UTR_MEDIATED_TRANSLATIONAL_REGULATION	104	0.27060822	2.0455742	0	0.17161362	0.351	9587	tags=77%, list=49%, signal=151%
REACTOME_TRANSLATION	144	0.22564332	1.9032145	0	0.19543718	0.707	9357	tags=69%, list=48%, signal=132%
**GO categories**								
DNA_DAMAGE_RESPONSESIGNAL_TRANSDUCTION_RESULTING_IN_INDUCTION_OF_APOPTOSIS	15	0.59700537	2.294397	0	0.10954069	0.108	3765	tags=73%, list=19%, signal=91%
REGULATION_OF_MITOSIS	41	0.3607356	2.1248572	0	0.14263822	0.363	3666	tags=51%, list=19%, signal=63%
DNA_DAMAGE_RESPONSESIGNAL_TRANSDUCTION	34	0.38482407	2.0864527	0.002155172	0.14303811	0.448	5121	tags=59%, list=26%, signal=80%
STEROID_BINDING	18	0.51839554	2.1577866	0	0.16232647	0.282	2729	tags=50%, list=14%, signal=58%
TRANSFERASE_ACTIVITY_TRANSFERRING_ALKYL_OR_ARYLOTHER_THAN_METHYLGROUPS	30	0.38621652	1.9660492	0	0.17542939	0.77	3754	tags=50%, list=19%, signal=62%
HEPARIN_BINDING	23	0.430639	1.9704957	0.015053763	0.19475801	0.759	5847	tags=70%, list=30%, signal=99%
STRUCTURAL_CONSTITUENT_OF_RIBOSOME	79	0.27841038	2.0030036	0.002314815	0.21167825	0.661	9714	tags=77%, list=50%, signal=154%
LIPID_METABOLIC_PROCESS	322	0.18721926	1.9066826	0	0.21296114	0.888	6340	tags=46%, list=33%, signal=67%
INDUCTION_OF_APOPTOSIS_BY_INTRACELLULAR_SIGNALS	24	0.41551518	1.9774078	0.006396588	0.21536268	0.742	3765	tags=54%, list=19%, signal=67%
CELL_CYCLE_CHECKPOINT_GO_0000075	47	0.3048062	1.8330754	0.006263048	0.21916085	0.971	3810	tags=45%, list=20%, signal=55%
NUCLEAR_HORMONE_RECEPTOR_BINDING	28	0.37142563	1.8867449	0.006185567	0.22293167	0.917	1585	tags=29%, list=8%, signal=31%
REGULATION_OF_DNA_REPLICATION	20	0.44011334	1.9125063	0.008179959	0.2272017	0.879	4859	tags=55%, list=25%, signal=73%
HORMONE_RECEPTOR_BINDING	29	0.3681721	1.8585308	0.011261261	0.2279066	0.957	1585	tags=28%, list=8%, signal=30%
PEROXISOME_ORGANIZATION_AND_BIOGENESIS	16	0.47301665	1.8697249	0.004115226	0.2280642	0.945	8042	tags=75%, list=41%, signal=128%
LIPID_BIOSYNTHETIC_PROCESS	97	0.24111676	1.8360211	0.002331002	0.22886348	0.97	3777	tags=38%, list=19%, signal=47%

SPHINGOLIPID_METABOLIC_PROCESS	28	0.3652482	1.8422383	0.006048387	0.23489282	0.968	4796	tags=50%, list=25%, signal=66%
GLYCOLIPID_METABOLIC_PROCESS	16	0.45011404	1.8054838	0.00814664	0.24744135	0.983	4486	tags=63%, list=23%, signal=81%
**KEGG categories**								
KEGG_PRIMARY_IMMUNODEFICIENCY	35	0.37557444	2.097092	0.00443459	0.067387976	0.081	5454	tags=63%, list=28%, signal=87%
KEGG_RIBOSOME	86	0.27904585	1.9882319	0	0.078129046	0.181	9587	tags=78%, list=49%, signal=153%
KEGG_DRUG_METABOLISM_CYTOCHROME_P450	69	0.27593297	1.904524	0.006976744	0.08724161	0.29	7951	tags=67%, list=41%, signal=113%
KEGG_PROPANOATE_METABOLISM	32	0.3215398	1.7396039	0.0125	0.13028204	0.636	4221	tags=44%, list=22%, signal=56%
KEGG_ASCORBATE_AND_ALDARATE_METABOLISM	23	0.38493398	1.7652289	0.026431719	0.1329639	0.572	5662	tags=61%, list=29%, signal=86%
KEGG_RETINOL_METABOLISM	61	0.27068487	1.7745104	0.008639309	0.15773137	0.552	6259	tags=56%, list=32%, signal=82%
KEGG_PORPHYRIN_AND_CHLOROPHYLL_METABOLISM	39	0.2943337	1.6745461	0.018907564	0.16795994	0.772	6104	tags=54%, list=31%, signal=78%

GSEA Analysis (Total mRNA)
Top 20 categories with FDR<0.25 shown
Sets downregulated following eIF4A1 knockdown

	SIZE	ES	NES	NOM p-val	FDR q-val	FWER p-val	RANK AT MAX	EADING EDGE
**Biocarta Categories**								
BIOCARTA_P53HYPOXIA_PATHWAY	22	-0.5545046	-2.2824137	0	0.05229664	0.042	635	tags=27%, list=3%, signal=28%
BIOCARTA_ATM_PATHWAY	20	-0.53155386	-2.1413314	0.001996008	0.08515919	0.137	2903	tags=55%, list=15%, signal=65%
BIOCARTA_TGFB_PATHWAY	19	-0.5239376	-2.0634	0.004255319	0.100633346	0.225	1335	tags=37%, list=7%, signal=40%
BIOCARTA_MTA3_PATHWAY	18	-0.51682776	-2.0150928	0.010351967	0.11334843	0.313	2219	tags=33%, list=11%, signal=38%
**Reactome categories**								
REACTOME_CHOLESTEROL_BIOSYNTHESIS	22	-0.5813942	-2.43895	0	0.012873751	0.026	2075	tags=41%, list=11%, signal=46%
REACTOME_TRANSPORT_OF_INORGANIC_CATIONS_ANIONS_AND_AMINO_ACIDS_OLIGOPEPTIDES	92	-0.37938014	-2.4614108	0	0.017679015	0.018	5072	tags=49%, list=26%, signal=66%
REACTOME_SPHINGOLIPID_DE_NOVO_BIOSYNTHESIS	30	-0.5027991	-2.3006651	0	0.032122694	0.09	368	tags=13%, list=2%, signal=14%
REACTOME_ENOS_ACTIVATION_AND_REGULATION	19	-0.5472194	-2.1584637	0.003929273	0.050877597	0.266	6540	tags=74%, list=34%, signal=111%
REACTOME_SIGNALING_BY_NOTCH	100	-0.32618147	-2.1730976	0	0.05525533	0.241	2450	tags=22%, list=13%, signal=25%
REACTOME_CELL_CELL_COMMUNICATION	117	-0.3087179	-2.1290076	0.001949318	0.05662337	0.332	5648	tags=48%, list=29%, signal=67%
REACTOME_METABOLISM_OF_LIPIDS_AND_LIPOPROTEINS	464	-0.22439192	-2.106108	0	0.058394272	0.378	3789	tags=28%, list=20%, signal=33%
REACTOME_AMINO_ACID_TRANSPORT_ACROSS_THE_PLASMA_MEMBRANE	30	-0.4668602	-2.1978102	0	0.05881011	0.206	500	tags=20%, list=3%, signal=20%
REACTOME_PHOSPHOLIPID_METABOLISM	191	-0.2577392	-2.0408244	0	0.0827846	0.52	1557	tags=16%, list=8%, signal=17%
REACTOME_PRE_NOTCH_EXPRESSION_AND_PROCESSING	42	-0.39084733	-2.0085554	0.006224067	0.08677245	0.612	2420	tags=21%, list=12%, signal=24%
REACTOME_GLYCEROPHOSPHOLIPID_BIOSYNTHESIS	82	-0.32294306	-2.0213702	0	0.08786809	0.581	6017	tags=46%, list=31%, signal=67%
REACTOME_SLC_MEDIATED_TRANSMEMBRANE_TRANSPORT	237	-0.23156886	-1.932178	0	0.11190721	0.796	5197	tags=40%, list=27%, signal=54%
REACTOME_REGULATION_OF_HYPOXIA_INDUCIBLE_FACTOR_HIF_BY_OXYGEN	23	-0.46264923	-1.943206	0.010245902	0.11196677	0.763	159	tags=13%, list=1%, signal=13%
REACTOME_TRANSMEMBRANE_TRANSPORT_OF_SMALL_MOLECULES	405	-0.21355653	-1.9450761	0	0.11990413	0.759	5074	tags=37%, list=26%, signal=49%
REACTOME_SIGNALING_BY_NOTCH1	68	-0.3213998	-1.9106721	0.001992032	0.12129831	0.849	2629	tags=24%, list=14%, signal=27%
REACTOME_ION_TRANSPORT_BY_P_TYPE_ATPASES	33	-0.39824012	-1.8884242	0.004040404	0.13151781	0.89	4935	tags=48%, list=25%, signal=65%
REACTOME_AMINO_ACID_AND_OLIGOPEPTIDE_SLC_TRANSPORTERS	48	-0.35493466	-1.840229	0.022312373	0.13467747	0.949	500	tags=15%, list=3%, signal=15%
REACTOME_FATTY_ACID_TRIACYLGLYCEROL_AND_KETONE_BODY_METABOLISM	167	-0.24466811	-1.8455423	0	0.13622907	0.941	3376	tags=27%, list=17%, signal=32%
REACTOME_NEPHRIN_INTERACTIONS	19	-0.46701908	-1.8476087	0.022222223	0.14170517	0.941	6030	tags=68%, list=31%, signal=99%
REACTOME_CELL_JUNCTION_ORGANIZATION	76	-0.3077681	-1.8515095	0.007633588	0.1464167	0.94	5204	tags=43%, list=27%, signal=59%
**GO categories**								
POSITIVE_REGULATION_OF_TRANSPORT	23	-0.5224196	-2.2090626	0	0.07202485	0.308	69	tags=13%, list=0%, signal=13%
REGULATION_OF_INTRACELLULAR_TRANSPORT	24	-0.52777135	-2.2165177	0.002118644	0.08186571	0.294	510	tags=17%, list=3%, signal=17%
REGULATION_OF_CYTOSKELETON_ORGANIZATION_AND_BIOGENESIS	30	-0.48455215	-2.2358496	0.00204918	0.08548269	0.252	2659	tags=43%, list=14%, signal=50%
REGULATION_OF_NUCLEOCYTOPLASMIC_TRANSPORT	21	-0.57155657	-2.250144	0.001984127	0.09755805	0.218	510	tags=19%, list=3%, signal=20%
PROTEIN_AMINO_ACID_O_LINKED_GLYCOSYLATION	18	-0.5546995	-2.146251	0	0.10403322	0.456	222	tags=22%, list=1%, signal=22%
NEGATIVE_REGULATION_OF_CELLULAR_COMPONENT_ORGANIZATION_AND_BIOGENESIS	28	-0.5375825	-2.3551	0	0.12036755	0.098	2531	tags=43%, list=13%, signal=49%
ACTIN_CYTOSKELETON	129	-0.31572586	-2.2647173	0	0.12621787	0.194	5554	tags=45%, list=29%, signal=63%
INDUCTION_OF_APOPTOSIS_BY_INTRACELLULAR_SIGNALS	24	-0.46525332	-1.9843404	0.004175365	0.137743	0.86	2306	tags=42%, list=12%, signal=47%
HEMATOPOIETIN_INTERFERON_CLASSD200_DOMAIN_CYTOKINE_RECEPTOR_ACTIVITY	33	-0.41156855	-1.9901904	0.002012072	0.13927013	0.849	3670	tags=48%, list=19%, signal=60%
MRNA_BINDING	23	-0.4776636	-1.9968852	0.006451613	0.14216901	0.839	724	tags=22%, list=4%, signal=23%
REGULATION_OF_ORGANELLE_ORGANIZATION_AND_BIOGENESIS	40	-0.40947926	-2.0584128	0.003984064	0.14390057	0.696	2659	tags=38%, list=14%, signal=43%
NEURON_APOPTOSIS	17	-0.5119384	-1.8810556	0.016842104	0.14462228	0.988	2310	tags=53%, list=12%, signal=60%
REGULATION_OF_TRANSPORT	64	-0.32663116	-1.8913227	0.002024292	0.14820041	0.982	562	tags=11%, list=3%, signal=11%
REGULATION_OF_LYMPHOCYTE_ACTIVATION	34	-0.3849829	-1.8816783	0.008438818	0.14838389	0.987	4557	tags=53%, list=23%, signal=69%
CORTICAL_CYTOSKELETON	20	-0.46843562	-1.894355	0.021367522	0.1507651	0.979	3797	tags=55%, list=20%, signal=68%
ECTODERM_DEVELOPMENT	80	-0.3170763	-1.9972401	0	0.15116654	0.837	2098	tags=25%, list=11%, signal=28%
EPIDERMIS_DEVELOPMENT	71	-0.35175005	-2.065177	0	0.15149452	0.68	2098	tags=28%, list=11%, signal=31%
DNA_REPLICATION	100	-0.28867814	-1.8688245	0.00209205	0.15222315	0.989	4255	tags=40%, list=22%, signal=51%
ACTIVE_TRANSMEMBRANE_TRANSPORTER_ACTIVITY	121	-0.28027177	-1.9327757	0	0.15247165	0.94	4647	tags=38%, list=24%, signal=50%
AMINE_TRANSMEMBRANE_TRANSPORTER_ACTIVITY	40	-0.37313342	-1.8820982	0.010351967	0.15260532	0.986	504	tags=15%, list=3%, signal=15%
**KEGG categories**								
KEGG_P53_SIGNALING_PATHWAY	68	-0.4378081	-2.5412247	0	0.003392452	0.003	2855	tags=44%, list=15%, signal=52%
KEGG_VIRAL_MYOCARDITIS	67	-0.36388394	-2.1886487	0	0.031090207	0.072	3526	tags=40%, list=18%, signal=49%
KEGG_SPHINGOLIPID_METABOLISM	37	-0.4508467	-2.2278397	0	0.03139731	0.051	446	tags=19%, list=2%, signal=19%
KEGG_GLYCEROPHOSPHOLIPID_METABOLISM	77	-0.34598848	-2.087179	0	0.036613315	0.141	1252	tags=19%, list=6%, signal=21%
KEGG_THYROID_CANCER	29	-0.47040305	-2.0984156	0	0.041465405	0.131	882	tags=24%, list=5%, signal=25%
KEGG_BLADDER_CANCER	42	-0.39755467	-2.040534	0.002012072	0.04370223	0.193	790	tags=17%, list=4%, signal=17%
KEGG_STEROID_BIOSYNTHESIS	17	-0.51668316	-1.9525689	0.008333334	0.07016209	0.325	1471	tags=35%, list=8%, signal=38%
KEGG_CELL_ADHESION_MOLECULES_CAMS	129	-0.25672847	-1.7782373	0.004	0.10134665	0.703	5139	tags=40%, list=27%, signal=54%
KEGG_FOCAL_ADHESION	196	-0.23696789	-1.8736428	0.002	0.10434793	0.481	5200	tags=42%, list=27%, signal=57%
KEGG_PATHOGENIC_ESCHERICHIA_COLI_INFECTION	55	-0.3288918	-1.8576694	0.004123712	0.10457591	0.525	4667	tags=42%, list=24%, signal=55%
KEGG_CHRONIC_MYELOID_LEUKEMIA	72	-0.29088187	-1.7797433	0.008080808	0.107617795	0.7	6451	tags=53%, list=33%, signal=79%
KEGG_LEUKOCYTE_TRANSENDOTHELIAL_MIGRATION	114	-0.26647222	-1.802328	0.001984127	0.10966305	0.643	5229	tags=45%, list=27%, signal=61%
KEGG_LYSOSOME	121	-0.25627694	-1.7860723	0.002070393	0.11153178	0.683	6920	tags=50%, list=36%, signal=78%
KEGG_RENAL_CELL_CARCINOMA	70	-0.2907536	-1.7463734	0.001869159	0.11319623	0.76	1176	tags=17%, list=6%, signal=18%
KEGG_MTOR_SIGNALING_PATHWAY	51	-0.32329378	-1.7318786	0.016701462	0.11439266	0.78	2217	tags=24%, list=11%, signal=26%
KEGG_NOTCH_SIGNALING_PATHWAY	47	-0.32627818	-1.7219028	0.012219959	0.1155542	0.804	1191	tags=15%, list=6%, signal=16%
KEGG_ECM_RECEPTOR_INTERACTION	83	-0.28989404	-1.8074018	0.005791506	0.116961285	0.636	2253	tags=25%, list=12%, signal=29%
KEGG_PATHWAYS_IN_CANCER	323	-0.19831394	-1.7094687	0.001818182	0.117387086	0.826	2317	tags=21%, list=12%, signal=23%
KEGG_GLYCEROLIPID_METABOLISM	49	-0.34926337	-1.8173573	0.014736842	0.12233589	0.617	1452	tags=22%, list=7%, signal=24%
KEGG_ADHERENS_JUNCTION	73	-0.27878734	-1.6785802	0.018828452	0.12639788	0.876	3626	tags=34%, list=19%, signal=42%
**miRNA Targets**								
TSCAN.ALL	10729	-0.1433729	-1.894173	0	0.006941582	0.054	4206	tags=24%, list=22%, signal=14%

Sets upregulated following eIF4A1 knockdown

	SIZE	ES	NES	NOM p-val	FDR q-val	FWER p-val	RANK AT MAX	EADING EDGE
**Biocarta categories**								
BIOCARTA_GSK3_PATHWAY	27	0.44091013	1.9561715	0.005825243	0.051351752	0.28	2949	tags=41%, list=15%, signal=48%
BIOCARTA_PITX2_PATHWAY	15	0.55091083	2.004263	0.002004008	0.058018893	0.196	1338	tags=27%, list=7%, signal=29%
BIOCARTA_MAPK_PATHWAY	85	0.30774373	1.9661897	0	0.05995498	0.262	2351	tags=29%, list=12%, signal=33%
BIOCARTA_LAIR_PATHWAY	17	0.54108113	2.036683	0.003703704	0.06967147	0.161	4176	tags=65%, list=22%, signal=82%
BIOCARTA_PROTEASOME_PATHWAY	28	0.44825545	2.055028	0.001945525	0.1197708	0.14	1512	tags=32%, list=8%, signal=35%
**Reactome categories**								
REACTOME_METABOLISM_OF_MRNA	208	0.3081843	2.5229495	0	9.22E-04	0.001	1512	tags=22%, list=8%, signal=24%
REACTOME_METABOLISM_OF_RNA	253	0.27851424	2.3438802	0	0.011401352	0.023	1167	tags=18%, list=6%, signal=19%
REACTOME_NONSENSE_MEDIATED_DECAY_ENHANCED_BY_THE_EXON_JUNCTION_COMPLEX	105	0.32517275	2.2775497	0	0.015829688	0.046	901	tags=17%, list=5%, signal=18%
REACTOME_ASSEMBLY_OF_THE_PRE_REPLICATIVE_COMPLEX	63	0.3372201	2.0847075	0.003838772	0.024177086	0.236	4001	tags=38%, list=21%, signal=48%
REACTOME_OLFACTORY_SIGNALING_PATHWAY	194	0.27149022	2.1973305	0	0.024698887	0.096	8305	tags=69%, list=43%, signal=120%
REACTOME_PEPTIDE_CHAIN_ELONGATION	84	0.32367435	2.090716	0	0.025808588	0.229	901	tags=17%, list=5%, signal=17%
REACTOME_JNK_C_JUN_KINASES_PHOSPHORYLATION_AND_ACTIVATION_MEDIATED_BY_ACTIVATED_HUMAN_TAK1	16	0.55516016	2.0658596	0.007407407	0.026316946	0.276	5949	tags=75%, list=31%, signal=108%
REACTOME_CDT1_ASSOCIATION_WITH_THE_CDC6_ORC_ORIGIN_COMPLEX	54	0.3750361	2.17059	0	0.02681107	0.128	4001	tags=41%, list=21%, signal=51%
REACTOME_GLUTATHIONE_CONJUGATION	23	0.49533388	2.0916848	0	0.028570406	0.229	5238	tags=70%, list=27%, signal=95%
REACTOME_RNA_POL_I_TRANSCRIPTION_TERMINATION	20	0.5096392	2.1040525	0.001879699	0.028662553	0.204	1641	tags=40%, list=8%, signal=44%
REACTOME_CDK_MEDIATED_PHOSPHORYLATION_AND_REMOVAL_OF_CDC6	46	0.39023116	2.109584	0	0.031597346	0.198	5991	tags=50%, list=31%, signal=72%
REACTOME_INFLUENZA_VIRAL_RNA_TRANSCRIPTION_AND_REPLICATION	100	0.29314867	1.9781009	0	0.033025455	0.49	901	tags=16%, list=5%, signal=17%
REACTOME_MAP_KINASE_ACTIVATION_IN_TLR_CASCADE	49	0.3876727	2.126775	0.001984127	0.033369616	0.179	5949	tags=57%, list=31%, signal=82%
REACTOME_VIF_MEDIATED_DEGRADATION_OF_APOBEC3G	49	0.35836902	1.9657505	0.003937008	0.033405803	0.53	1512	tags=27%, list=8%, signal=29%
REACTOME_3_UTR_MEDIATED_TRANSLATIONAL_REGULATION	104	0.29209486	1.9973259	0	0.033793166	0.443	901	tags=17%, list=5%, signal=18%
REACTOME_DESTABILIZATION_OF_MRNA_BY_AUF1_HNRNP_D0	50	0.3642544	2.025023	0.003883495	0.03430339	0.37	1609	tags=28%, list=8%, signal=30%
REACTOME_REGULATION_OF_MRNA_STABILITY_BY_PROTEINS_THAT_BIND_AU_RICH_ELEMENTS	80	0.3140882	2.0011885	0	0.03447029	0.429	1609	tags=25%, list=8%, signal=27%
REACTOME_RNA_POL_I_TRANSCRIPTION_INITIATION	23	0.4562375	1.9673575	0.001801802	0.034532376	0.521	1641	tags=35%, list=8%, signal=38%
REACTOME_RESPIRATORY_ELECTRON_TRANSPORT	63	0.32634	1.978862	0.004048583	0.034554277	0.488	2010	tags=27%, list=10%, signal=30%
REACTOME_MAPK_TARGETS_NUCLEAR_EVENTS_MEDIATED_BY_MAP_KINASES	30	0.42440772	1.9837806	0.003831418	0.03473117	0.467	4739	tags=53%, list=24%, signal=70%
**GO categories**								
MITOCHONDRIAL_MEMBRANE	85	0.34837475	2.2734764	0	0.014455031	0.07	2498	tags=29%, list=13%, signal=34%
PATTERN_SPECIFICATION_PROCESS	31	0.49074876	2.3115706	0	0.01500384	0.055	1112	tags=32%, list=6%, signal=34%
MITOCHONDRIAL_ENVELOPE	96	0.328585	2.184427	0	0.017642343	0.163	2561	tags=29%, list=13%, signal=33%
SPLICEOSOME	51	0.39874312	2.2285132	0	0.018309217	0.108	904	tags=16%, list=5%, signal=16%

STRUCTURAL_CONSTITUENT_OF_RIBOSOME	79	0.37299684	2.3721433	0	0.018444264	0.022	964	tags=20%, list=5%, signal=21%
SMALL_NUCLEAR_RIBONUCLEOPROTEIN_COMPLEX	22	0.52627647	2.1875756	0	0.019588683	0.159	357	tags=18%, list=2%, signal=19%
MITOCHONDRIAL_INNER_MEMBRANE	66	0.3924545	2.3291326	0	0.020505786	0.05	2010	tags=30%, list=10%, signal=34%
ORGANELLE_INNER_MEMBRANE	74	0.3552572	2.1936674	0	0.02177734	0.152	2010	tags=28%, list=10%, signal=32%
MITOCHONDRIAL_PART	140	0.2832095	2.0633895	0	0.04590445	0.436	2561	tags=27%, list=13%, signal=31%
NUCLEOLAR_PART	17	0.53578246	2.0710597	0.007722008	0.047790132	0.413	545	tags=29%, list=3%, signal=30%
RIBONUCLEOPROTEIN_COMPLEX	141	0.26861376	2.0103428	0	0.0658992	0.595	1110	tags=15%, list=6%, signal=16%
MITOCHONDRION	335	0.21580894	1.9687381	0	0.08563331	0.73	2102	tags=21%, list=11%, signal=23%
HISTONE_DEACETYLASE_COMPLEX	20	0.4627358	1.915685	0	0.09782719	0.873	6387	tags=70%, list=33%, signal=104%
METHYLTRANSFERASE_ACTIVITY	36	0.39108592	1.9164327	0.001901141	0.103223994	0.869	2271	tags=33%, list=12%, signal=38%
RECEPTOR_SIGNALING_PROTEIN_SERINE_THREONINE_KINASE_ACTIVITY	33	0.40348503	1.9347781	0.005952381	0.10359031	0.839	1236	tags=24%, list=6%, signal=26%
TRANSFERASE_ACTIVITY_TRANSFERRING_ONE_CARBON_GROUPS	37	0.37840745	1.9244865	0.005813954	0.10360596	0.859	2271	tags=32%, list=12%, signal=37%
TRANSLATION	179	0.23443417	1.8795023	0.001949318	0.12085544	0.928	3679	tags=28%, list=19%, signal=35%
PROTEIN_SERINE_THREONINE_KINASE_ACTIVITY	200	0.229881	1.8707961	0	0.12143372	0.942	3818	tags=31%, list=20%, signal=38%
TRANSCRIPTION_REPRESSOR_ACTIVITY	148	0.24085638	1.8375266	0	0.148141	0.963	1511	tags=17%, list=8%, signal=18%
**KEGG categories**								
KEGG_PROTEASOME	44	0.39340857	2.1157224	0.001872659	0.01766739	0.067	1512	tags=30%, list=8%, signal=32%
KEGG_OXIDATIVE_PHOSPHORYLATION	115	0.30659038	2.1783392	0	0.018358644	0.047	2010	tags=25%, list=10%, signal=28%
KEGG_RNA_POLYMERASE	29	0.46898487	2.1843812	0.003773585	0.035198532	0.045	847	tags=28%, list=4%, signal=29%
KEGG_RIBOSOME	86	0.29196167	1.9068103	0	0.06488873	0.284	901	tags=15%, list=5%, signal=16%
KEGG_HUNTINGTONS_DISEASE	170	0.23834488	1.8654752	0	0.06960748	0.361	1688	tags=21%, list=9%, signal=23%
KEGG_OLFACTORY_TRANSDUCTION	246	0.21558295	1.8294756	0	0.07608218	0.444	8238	tags=64%, list=42%, signal=110%
KEGG_PARKINSONS_DISEASE	112	0.25967684	1.7955871	0.006198347	0.082040206	0.531	3663	tags=30%, list=19%, signal=37%
KEGG_BASAL_CELL_CARCINOMA	55	0.31662443	1.7745699	0.006036217	0.085056245	0.589	785	tags=15%, list=4%, signal=15%
KEGG_PURINE_METABOLISM	155	0.23145445	1.7556967	0.002087683	0.08526107	0.639	4471	tags=32%, list=23%, signal=41%
KEGG_PYRIMIDINE_METABOLISM	95	0.25966406	1.7140204	0.00625	0.091283806	0.732	847	tags=15%, list=4%, signal=15%
KEGG_SPLICEOSOME	122	0.23903011	1.7262952	0.00591716	0.09319797	0.709	1397	tags=14%, list=7%, signal=15%
KEGG_PANTOTHENATE_AND_COA_BIOSYNTHESIS	16	0.43753055	1.641037	0.044198897	0.13326482	0.88	7199	tags=69%, list=37%, signal=109%
KEGG_NUCLEOTIDE_EXCISION_REPAIR	44	0.29050308	1.543092	0.056285176	0.20406431	0.973	9322	tags=68%, list=48%, signal=131%
KEGG_PROXIMAL_TUBULE_BICARBONATE_RECLAMATION	22	0.37140813	1.5542997	0.060037524	0.20595537	0.966	6604	tags=55%, list=34%, signal=83%
KEGG_CYTOSOLIC_DNA_SENSING_PATHWAY	56	0.26836112	1.5158098	0.060491495	0.22371818	0.986	8580	tags=64%, list=44%, signal=115%
KEGG_RIG_I_LIKE_RECEPTOR_SIGNALING_PATHWAY	70	0.24301048	1.4873179	0.036893204	0.2475609	0.992	7947	tags=56%, list=41%, signal=94%
KEGG_ONE_CARBON_POOL_BY_FOLATE	17	0.3650509	1.4104551	0.11676083	0.24797313	0.999	1148	tags=18%, list=6%, signal=19%
**miRNA Targets**								
TSCAN.ALL	10729	-0.14179148	-1.782787	0	0.007799148	0.133	3316	tags=20%, list=17%, signal=11%
