# Supplementary material for: The malignant phenotype in breast cancer is driven by eIF4A1-mediated changes in the translational landscape
Source: Cell Death Dis. 2015 Jan 22;6(1):e1603–. doi: 10.1038/cddis.2014.542 (PMC4669741; doi:10.1038/cddis.2014.542)
Supplement: Supplementary Data [file cddis2014542x4.doc]

**List of supplementary data**

**Supplementary Table 1.** Summary of immunohistochemical data.

**Supplementary Table 2.** Univariate survival models.

**Supplementary Table 3.** Correlations between translation initiation factors and oestrogen receptor expression.

**Supplementary Table 4A**. Associations between markers and clinicopathological data within the type of disease (ER-positive or ER-negative) in which the marker has prognostic value.

**Supplementary Table 4B.** Remaining associations between markers and clinicopathological data (i.e. in the disease type lacking prognostic value for the variable).

**Supplementary Table 5.** eIF4A1-dependent and independent transcripts.

**Supplementary Table 6.** Analysis of 5’ UTR characteristics of translationally eIF4A1-dependent and independent mRNAs.

**Supplementary Table 7.** Over-representation and Gene Set Enrichment analyses of eIF4A1-dependence at the level of translation (polysome/subpolysome redistribution) and total mRNA abundance.

**Supplementary Table 8.** Details of immunohistochemical assays.

**Supplementary Table 9.** Numbers at risk in Kaplan-Meier analyses.

**Supplementary Figure 1.** Correlation of RNASeq and microarray data.

**Supplementary Figure 2.** Loss of immunohistochemical positivity in siRNA-treated cells demonstrates the specificity of the antibody assays. **Supplementary Figure 3.** Ranked list of eIF4A1-dependent and -independent mRNAs.
